# Multistate, Polarizable QM/MM Embedding Scheme Based on the Direct Reaction Field Method: Solvatochromic Shifts, Analytical Gradients and Optimizations of Conical Intersections in Solution

**DOI:** 10.1021/acs.jctc.3c01018

**Published:** 2024-02-08

**Authors:** Alexander Humeniuk, William J. Glover

**Affiliations:** † NYU Shanghai, 567 West Yangsi Road, Shanghai 200124, China; ‡ NYU-ECNU Center for Computational Chemistry at NYU Shanghai, 3663 Zhongshan Road North, Shanghai 200062, China; § Shanghai Frontiers Science Center of Artificial Intelligence and Deep Learning, NYU Shanghai, 567 West Yangsi Road, Shanghai 200124, China; ∥ Department of Chemistry, New York University, New York, New York 10003, United States

## Abstract

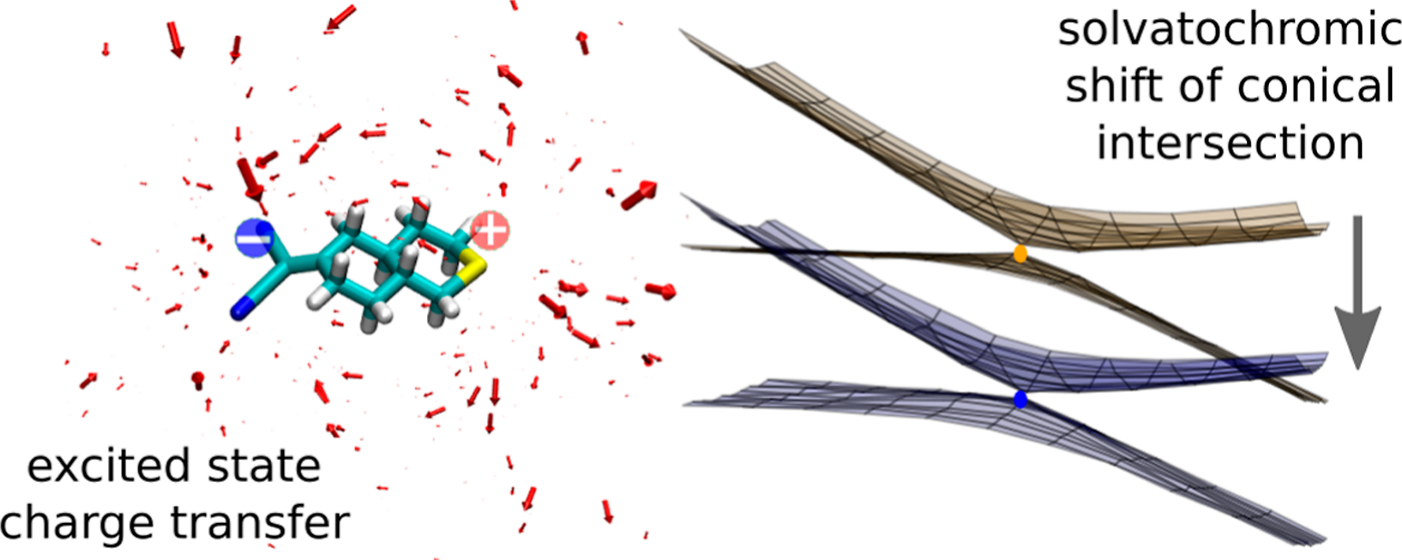

We recently introduced a polarizable embedding scheme based on an integral-exact reformulation of the direct reaction field method (IEDRF) that accounts for the differential solvation of ground and excited states in QM/MM simulations. The polarization and dispersion interactions between the quantum-mechanical (QM) and molecular-mechanical (MM) regions are described by the DRF Hamiltonian, while the Pauli repulsion between explicitly treated QM electrons and the implicit electron density around MM atoms is modeled with effective core potentials. A single Hamiltonian is used for all electronic states so that Born–Oppenheimer states belonging to the same geometry are orthogonal and state crossings are well-defined. In this work, we describe the implementation of the method using graphical processing unit acceleration in TeraChem, where it is combined with multiple electronic structure methods, including Hartree–Fock, time-dependent density functional theory, and complete active space self-consistent field. In contrast with older implementations of the DRF method, integrals of the polarization operators are evaluated exactly. Expressions for ingredients needed to construct analytical gradients and nonadiabatic coupling vectors are derived and tested by optimizing a conical intersection between two excited states in the presence of a polarizable solvent shell. The method is applied to estimate the solvent shifts of absorption energies of a series of donor–acceptor dyes having low-lying charge-transfer states. Even for a nonpolar solvent such as *n*-hexane, the inclusion of its static polarizability leads to non-negligible shifts that improve the agreement to essentially quantitative levels (0.03 eV) with full-system calculations. Good agreement with the positions of the experimental absorption maxima measured in solution is also observed.

## 1 Introduction

Photochemical reactions can often be understood by concentrating on relatively small molecular components, the chromophores, which are responsible for the absorption of light. Light-induced processes of technological interest, or those occurring in nature such as photosynthesis,^1^ happen in the condensed or liquid phase, where the environment affects the photophysical properties of the chromophores. Some solvent effects can be understood by modeling the solvent as a structureless dielectric continuum^2−5^ or viscous medium: the color of some chromophores changes depending on the solvent polarity^6^ and aggregation induced emission^7^ is observed when nonradiative deactivation channels are blocked mechanically by the surrounding molecules. However, other solvent effects defy simple explanations in terms of macroscopic solvent properties or steric hindrance and require an atomistic model. A particularly important example is the unidirectional charge separation in certain photosynthetic systems, such as the purple bacteria reaction center.^8^ These protein complexes contain two identical branches of pigments, but only one of them is active. Although the electronic excitation is strictly localized to the chromophores, the functioning of the reaction center is finely tuned by the protein matrix. Several theoretical^9,10^ and experimental^11,12^ studies have concluded that the asymmetry, which directs the charge transfer exclusively along the active branch, is produced by the unequal dielectric screening in the protein matrix. Since the protein complex contains thousands of atoms, a fully quantum-mechanical treatment is out of the question. This motivates the division of such complex systems into a small part treated with quantum mechanics (QM) and a larger environment treated with a classical molecular mechanics (MM) force field.^13^ The coupling between the two systems can be decomposed into electrostatic and steric interactions, as well as polarization and dispersion. Although the electrons in the MM part are not explicitly accounted for, one must not forget that all of these interactions are quantum-mechanical in nature: steric effects arise from Pauli repulsion, and static polarization and dispersion are the result of the correlated motion of electrons in both the MM and QM part. Classical approximations for the coupling in the form of pairwise Lennard-Jones potentials, fixed point charges, and polarizable force fields do not necessarily carry over without adjustments to the situation where the QM region is electronically excited.

While QM/MM simulations with polarizable embedding schemes are routinely performed for ground-state reactions, the extension to excited states is complicated by the fact that different electronic states react differently to the polarizable environment.^14^ Charge-transfer states, for instance, induce a reaction field much larger than that of local excitations. The polarizability of a molecule, and with it the dispersive attraction to the surrounding MM region, also changes in the excited state.

It is important to note that in polar solvents most of the dielectric response to long-lived excited states comes from the slow reorientation of the solvent molecules, which could be captured by running electrostatic embedding QM/MM molecular dynamics simulations in the excited state. What this simple QM/MM approach misses is the screening due to the instantaneous reaction of the environment’s electrons to the new charge distribution in the excited state. Macroscopically, this instantaneous polarization is quantified by the high-frequency dielectric constant, which for most saturated organic solvents amounts to approximately ϵ_∞_ ≈ 2.0 and does not vary among solvents as much as the static (or zero-frequency) dielectric constant (e.g., ϵ_r_ = 1.8 for *n*-hexane vs ϵ_r_ = 78 for water). However, in apolar solvents, the dielectric screening is predominantly due to the fast response of the electrons. Even then, the Coulomb interaction  is only about half as strong as in a vacuum, and the stabilization of long-range charge transfer in the excited state can be very large.

In standard polarizable QM/MM approaches, only a single electronic state (usually the ground state) is considered, and the point dipoles in the MM region are induced by the mean field arising from the electron density and the fields from all classical point multipoles.^14^ The induced dipoles are determined by minimizing the total electronic energy of the combined QM and polarizable MM subsystems including the work required to create the dipoles. Since the electron density and induced dipoles mutually depend on each other, the electronic structure and electrostatic problem are intertwined and have to be solved self-consistently. Effectively this introduces a complicated dependence of the QM Hamiltonian on the electron density. While this does not pose a conceptual problem for a single electronic state, extending the mean-field solvation approach to multiple electronic states is fraught with inconsistencies because it is not clear how the mean field should be obtained. Possible choices, none of which are entirely satisfactory, are the mean field arising from each state’s density one at a time (i.e., state-specific solvation discussed below), the average density of multiple electronic states of interest, or the density of the ground state.

Previous efforts to combine polarizable embedding schemes with excited-state calculations can be roughly divided into linear-response^9,15,16^ and state-specific formulations.^17−20^ In linear-response approaches to finding electronic excitations, the ground state and excited states are not treated on the same footing. In a first step, the reaction field of the induced dipole moments is determined self-consistently with the charge distribution of the ground-state wave function. In the second step, electronic excitation energies are determined from the response of the electronic and dipole degrees of freedom to an oscillating electric field. When the response states are solved for in the second step, the reaction of the induced dipoles to the transition density of the response state is retained to linear order. As pointed out for the related polarizable continuum models (PCM), linear response then recovers only a dispersion-like portion of the solvation’s influence on the excitation energy,^21^ and the solvent response to long-range (dark) CT states is largely missed.^22^ As a result, linear-response polarization tends to overestimate photoexcited CT-state energies.

In contrast to linear response, state-specific approaches^17−20^ determine the polarization response self-consistently for a specific electronic state. This idea appears to originate with a similar approach used for PCM^23^ and even earlier in analytic solvation models.^24^ A separate calculation must be performed for each state. Since the Hamiltonian depends on the wave function through the induced dipole moments, different electronic states are not eigenfunctions of the same Hamiltonian anymore. Therefore, transition matrix elements and state crossings become ill-defined.^18,25,26^ In addition, root-flipping issues are commonly observed, particularly between states that are close in energy.^18,26^

In a previous paper,^26^ we showed that an early polarizable embedding method called direct reaction field (DRF),^27,28^ which, although seems to have fallen out of use, overcomes the problems of both linear-response and state-specific solvation models and allows one to obtain several excited states in a single run. This section briefly reviews the history of the DRF^27^ and dipole interaction models.^29^ In one of the earliest mixed quantum-classical simulations of an enzymatic reaction, Warshel and Levitt already realized the importance of the polarizable environment for stabilizing reaction intermediates.^30,31^ They represented the solvent molecules as a collection of polarizable dipoles that respond to the charge distribution of the substrate. The effective polarizability of such an ensemble of interacting dipoles can be estimated from atomic parameters using Applequist’s model.^29^ Based on these initial developments, the DRF method was invented by Thole and van Duijnen in the early 1980s.^27,28^ They considered a system divided into a quantum-mechanical region and an atomistic environment of point charges and interacting polarizable dipoles. The starting point is the classical expression for the polarization energy of the dipoles, , where ***f*** are the electric fields created by the QM charge distribution and ***A*** is the effective polarizability of the environment. By replacing the electrostatic fields, ***f***, with their quantum-mechanical equivalents, ***f̂***, which depend on the electronic coordinates, one arrives at a quantum-mechanical operator for the polarization energy.

The approach is called the direct reaction field in contrast to the self-consistent reaction field. In the self-consistent reaction field, another name for state-specific solvation, the response of the solvent is introduced only after determining the solute wave function and charge distribution, which then creates a field that acts back on the solute. Since the electric fields enter the polarization energy in the form of an expectation value, , the Hamiltonian acquires a complicated nonlinear dependence on the electronic state of interest.^27^ In the DRF method, on the other hand, the reaction field is added directly in the form of a polarization Hamiltonian. Crucially, there are no nonlinear terms, as in the self-consistent reaction field method, and the same Hamiltonian is used for all electronic states. Thus, solvent effects are incorporated directly into the Hamiltonian matrix elements such that the polarizable solvent molecules effectively screen the Coulomb interactions between all charged solute particles. The polarization Hamiltonian alters both the one- and two-electron operators and thus introduces additional correlations between electrons through their interaction with the solvent. The polarization energy is determined in the usual way as an expectation value of the polarization Hamiltonian, .^27^ Due to the assumption of an instantaneous response of the induced MM dipoles to the electronic coordinates of the QM region, the method also approximately captures correlations between the electrons and MM dipoles, i.e., dispersion interactions between QM and MM particles, discussed in detail in our previous paper.^26^

DRF has seen a number of applications over the years, some of which are summarized in ref (32). While initial applications of the method focused on the electronic ground state, DRF was used to compute solvatochromic shifts of the π* ← n transition of acetone in various solvents.^33^ Random conformations were sampled from a Monte Carlo simulation using a classical force field to average the solvent degrees of freedom. The shifts were estimated from two self-consistent field calculations: a restricted HF calculation for the ground state S_0_ and a restricted open-shell calculation for the S_1_ state. The blue shift in the polar solvents was predicted to be in good agreement with the experimental data.

Since DRF provides an atomistic description of the solvent, it is suitable to investigate situations where the interaction with the environment breaks the symmetry of degenerate excited states. An asymmetric solvent shell can break two charge resonances with no net dipole moment into charge-separated states with large dipole moments.^34,35^ Grozema and Van Duijnen studied the relaxation of the S_1_ state of bianthryl in solution with the help of semiempirical configuration interaction combined with DRF.^35^ They showed that even nonpolar solvents can create considerable local electric fields that fluctuate with the reorientation of the solvent molecules but cancel on average.^35^ The DRF method has also been applied to the study of excitations in solids using the embedded cluster approach.^36^

If there is no quantum-mechanical region, DRF turns into a polarizable force field, which goes by the name discrete reaction field and unfortunately has the same acronym as DRF.^37−40^ The discrete reaction field itself has a polarizable QM/MM extension, which treats the solvent atomistically, but takes the expectation value of the electric fields arising in the QM region to polarize the MM region and therefore formally requires a self-consistent solution.^32^ The discrete reaction field idea has also been extended to a form of polarizable continuum model, which may be combined with a polarizable atomistic treatment of some of the solvent molecules.^32^

Although the direct reaction field method is in some sense simpler than self-consistent solvation models, it has not been widely adopted. This might be due to difficulties in the implementation. Technical details of the implementation of DRF can be found in ref (41). The additional matrix elements of the polarization operators were evaluated as Taylor expansions around arbitrary atomic centers. While this simplifies the resulting expressions, this can introduce symmetry-breaking artifacts^32^ and seems to preclude the evaluation of analytical gradients of the energy.

In a previous study, we described a preliminary implementation of DRF that was limited to neutral atomic solvents and Hartree–Fock (HF) and complete active space self-consistent field (CASSCF) electronic structures.^26^ Building on this previous study, the aim of this work is to revive the direct reaction field method by making a number of technical improvements: (a) All integrals are evaluated exactly using a special library for polarization integrals.^42,43^ Therefore, matrix elements of the polarization Hamiltonian are smooth functions of the nuclear coordinates. (b) TeraChem is chosen as a development platform because of its clever abstraction of molecular integral routines and GPU acceleration.^44^ This code formulates many electronic structure methods in terms of core Hamiltonian and Coulomb and exchange operators applied to generalized density matrices (“J- and K-builds”). By modifying only those few integral routines, many quantum-chemistry methods such as HF, density functional theory (DFT), time-dependent density functional theory (TD-DFT), configuration interaction singles (CIS), and CASSCF can be combined straightforwardly with the explicit solvent model provided by DRF. (c) Expressions for the analytical gradients of the solvation energy are derived. In TeraChem, the gradients of the energy or the nonadiabatic coupling vectors are expressed as contractions of derivatives of the core Hamiltonian and the J- and K-operators with generalized density matrix-like objects. Similar to energies, modification of a handful of routines then provides analytical gradients for all of the mentioned electronic structure methods in combination with DRF. To reflect these developments, we call the method integral-exact direct reaction field (IEDRF). We demonstrate the method for vertical excitation energy calculations with QM/MM-IEDRF embedding and conical intersection optimizations in a polarizable environment.

The rest of the article is structured as follows: First the basic equations of the direct reaction field are rederived (Section 2.1) and then reformulated with analytical integrals and effective Coulomb and exchange operators (Section 2.2). Then, the ingredients for assembling analytical gradients are worked out (Section 2.3). As a proof of principle, solvatochromic shifts of a series of bichromophoric dyes in *n*-hexane are computed at the TD-DFT level of theory (Section 3.1). The minimum-energy conical intersection between two excited states of one of the dyes is optimized in a shell of polarizable solvent molecules (Section 3.2). The polarizable embedding method is verified by comparison with DFT calculations where a large number of solvent molecules are included in the QM region (Section 3.3). Finally, the scaling of the method, convergence with the system size, and its computational cost are explored in Section 3.4. The article concludes with some justifications for combining DRF with DFT (Section 4).

## 2 Theoretical Methods

Atomic units are used throughout.

### 2.1 Polarization Hamiltonian

The distribution of all free charges in the whole system is denoted by
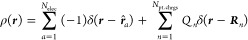
1The first term corresponds to the electrons, while the second term captures all *N*_pt.chrgs_ = *N*_QM_ + *N*_MM_ classical point charges, of which there are *N*_QM_ QM nuclei with a charge equal to the atomic number, *Q*_*n*_ = *Z*_*n*_, as well as *N*_MM_ MM atoms with partial charges.  is the quantum-mechanical position operator for electron *a*, while ***R***_*n*_ is the position vector of a classical particle. In addition to charged particles, a number of polarizable sites are included in the MM region. Not all MM atoms will be made polarizable in order to save computational time, and typically, we include polarizable sites on only the first few solvation shells around the QM region. Therefore, it is preferable to keep separate counts of the point charges and the polarizable atoms denoted by indices *i* and *j* (running over 1, ..., *N*_pol_).

An electric field, ***E***, induces a dipole, ***p***_*i*_, proportional to the atomic dipole polarizability, α_*i*_, of the atom
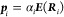
2The induced dipole itself also generates an electrostatic field, which can induce other dipoles. Therefore, if there is more than one polarizable atom, the total electric field that enters eq 2 consists of two parts: (1) The electric field, ***f***_*i*_[ρ], generated by the free charges, ρ(***r***), and (2) the electric field generated by the induced dipoles
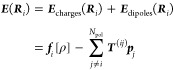
3Here, ***T***^(*ij*)^ is the dipole field tensor,^29^ of dimensions 3 × 3 × *N*_pol_ × *N*_pol_, which describes the electrostatic interaction between point dipoles.^45,46^ When two point dipoles come closer than the typical extension of an atom, the dipole field tensor is damped to avoid the polarization catastrophe.^45^ We refer the reader to the Supporting Information for expressions of ***T***^(*ij*)^ and the damping functions.

Combining the electric fields into a supervector, the operator for the polarization energy can be expressed as a quadratic form in ***f***
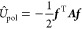
4where ***A*** is the effective dipole polarizability of the MM system (see Supporting Information for details)
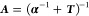
5It is important to note that  is a many-electron operator. The fields depend on the quantum mechanical operator for the electronic charge density and are therefore functions of the instantaneous positions of all electrons, . This sets the direct reaction field apart from mean-field solvation models, where ***f*** depends on the expectation value of the electronic charge density.

### 2.2 Modified One- and Two-Electron Integrals

In the DRF formalism, the presence of polarizable atoms modifies the Hamiltonian of the QM system. It changes not only the one-electron part of the Hamiltonian, as external point charges or traditional self-consistent polarizable embedding does, but also the two-electron part. In this way, the DRF method accounts for screening effects induced by a polarizable environment. In this section, the corrections to the two-electron, one-electron, and constant parts of the Hamiltonian are derived.

For convenience, we tabulate some of the frequently used symbols and index conventions in Table 1.

**Table 1:** Frequently Used Symbols and Index Conventions

symbol	description
ρ(***r***)	total charge density
*a*, *b*	indices for *N*_elec_ electrons
*m*, *n*	indices for *N*_pt.chrgs_ point charges (nuclei and MM charges)
*i*, *j*	indices for *N*_pol_ polarizable atoms
α, β	enumerate Cartesian components of three-dimensional space
μ, ν, λ, σ, γ, δ	indices for *N*_AO_ atomic orbitals
*Q*_*n*_	nuclear/MM charge of atom *n*
***p***_*i*_	induced dipole moment at polarizable atom *i*
***f*** = (***f***_1_, ..., ***f***_*N*pol_)	supervector of electric fields from free charges at polarizable atoms
***f***_*i*_	electric field from free charges at polarizable atom *i*
***A***	supermatrix of effective dipole polarizabilities of MM region
***f̂***^(*e*)^	supervector of electric field from all electrons at polarizable atoms
***f̂***_***i***_^(*e*)^	electric field from all electrons at polarizable atom *i*
(−1)***f̂***_*ia*_^(*e*)^	electric field from electron *a* at polarizable atom *i*
***f***^(*n*)^	supervector of electric fields from all point charges at polarizable atoms
***f***_*i*_^(*n*)^	electric field from all point charges at polarizable atom *i*
*Q*_*n*_***f***_*in*_^(*n*)^	electric field from point charge *n* at polarizable atom *i*
***F***_μν_^(*e*)^	supervector of matrix elements of an electron’s damped electric field at polarizable atoms in AO basis
***F***_*i*,μν_^(*e*)^	matrix element of an electron’s damped electric field at polarizable atom *i* in AO basis
***F***^(*n*)^	supervector of point charges’ damped electric field at polarizable atoms
***F***_*i*_^(*n*)^	point charges’ damped electric field at polarizable atom *i*
*I*_μν_^αβ^(***R***_*i*_)	αβ Cartesian component of core-polarization potential matrix element for polarizable atom *i*

Following eq 1, the electric field created by the free charges on a polarizable atom at ***R***_*i*_ is
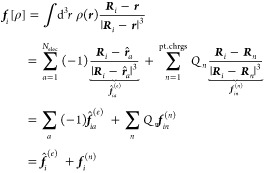
6In the last equation, the contributions from the electrons and those from the points charges have been separated. The field due to the electrons is an electronic operator (denoted by the hat). Putting the field into eq 4 gives the polarization Hamiltonian, which has to be added to the Hamiltonian of the QM system
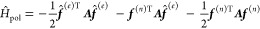
7After grouping the terms into two-electron and one-electron operators and zero-electron terms, the polarization Hamiltonian reads
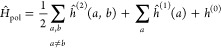
8The two-electron operator accounts for the additional interaction between two different electrons via the polarizable sites
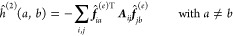
9The one-electron operator contains the interactions between nuclei and electrons as well as the self-interactions of the electrons, which are mediated by the polarizable sites
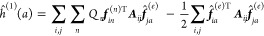
10The term involving only nuclei and point charges is a (geometry dependent) constant
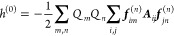
11that includes both the polarization contribution from the QM nuclei as well as the polarization energy of the MM region.

Now, we wish to know the representation of these operators in a basis of atom-centered Gaussian-type orbitals μ(***r***), ν(***r***), λ(***r***), and σ(***r***). Strictly speaking, integrals of the type ∫d^3^*r* μ(***r***)|***r*** – ***R***_*i*_|^–*k*^ν(***r***) do not exist if *k* > 2 because of the singularity of the polarization operator at ***r*** = ***R***_*i*_. The singularity is, however, not physical and arises from the treatment of MM-polarizable atoms as point-induced dipoles, when in reality they should have a finite charge distribution. The polarization operator should then be damped at a short range. To model this effect, a damping function
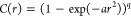
12is included, which ensures the existence of all polarization integrals. With the damping function, the matrix elements of an electron’s electric field at ***R*** is written as
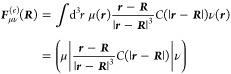
13The individual components
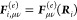
14with *i* = 1, ..., *N*_pol_ are combined into a supervector of dimension 3 × *N*_pol_
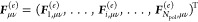
15for each combination of atomic orbitals μ, ν. The electric fields generated by the nuclei and MM point charges at the position of a polarizable atom ***R***_*i*_
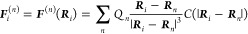
16are similarly combined into a supervector of size 3*N*_pol_
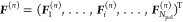
17It is important that the same damping function, *C*(*r*) (defined in eq 12), is used for the electronic and nuclear fields, so that for a neutral molecule the fields cancel appropriately at short and intermediate ranges.

Partial charges on MM atoms are usually optimized to reproduce the correct electrostatic potential on the surface of the molecule, while the electric fields generated by them inside the molecule are not physically meaningful. Therefore, one has to be careful in excluding monopole fields from point charges on MM atoms that are directly bonded to a polarizable atom. MM force fields keep an exclusion list to remove nonbonded interactions between certain atoms. The same list is used to exclude point charge *n* from the summation in eq 16 if it belongs to the exclusion list of the polarizable atom at ***R***_*i*_.

For a given geometry, vectors ***F***_μν_^(*e*)^ and ***F***^(*n*)^ are calculated once and stored in memory. The memory requirements for this are *N*_AO_^2^ × 3 × *N*_pol_ + 3 × *N*_pol_. All matrix elements of the two- and zero-electron operators can be assembled from this information as well as the first term of the one-electron operator (eq 10). However, the one-electron operator also contains a new type of four-center integral. This integral arises from the second term of eq 10 and has the form
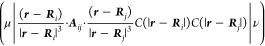
18If *i* and *j* refer to the same polarizable site, the integral reduces to a sum of one-electron integrals
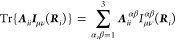
19α, β = 1, 2, 3 enumerate the elements of the 3 × 3 matrix ***A***_*ii*_ and the additional one-electron integrals  take the form of core polarization potentials.^47^ They are defined as
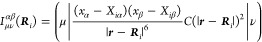
20with  and . While the electric field integrals in eq 13 do not necessarily require a damping function, the integrals of eq 20 would not exist without it. For consistency, the damping function has been included in both expressions.

If there is only a single polarizable site with polarizability α_*i*_ then the effective polarizability equals the atomic dipole polarizability, which is isotropic, ***A*** = **α** = diag(α_*i*_, α_*i*_, α_*i*_), and expression 19 simplifies to
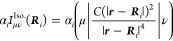
21

For the integrals *F*_*i*,μν_^α(*e*)^ and *I*_μν_^αβ^ defined by eqs 14 and 20, respectively, analytical expressions exist. Integrals of this type were solved for the first time by Schwerdtfeger^48^ and have been implemented recently by us in an open-source library.^42,43^ However, we are not aware of an analytical solution for the multisite case in eq 18 (when *i* ≠ *j*), and we therefore approximate these integrals by a resolution-of-identity trick (note: the original DRF method uses a similar trick, but in the molecular orbital basis).^32^ The scalar product in eq 18 is split by inserting the identity
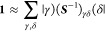
22where *S*_γδ_ = (γ|δ) is the overlap matrix. In principle, a larger auxiliary basis could be used in the resolution of identity; however, we found that sufficient accuracy was obtained using the primary basis set. If diffuse orbitals are present, then the overlap matrix might be singular. In this case, the inverse has to be replaced with the pseudoinverse, where small singular values below a certain threshold have been removed. Equation 18 becomes
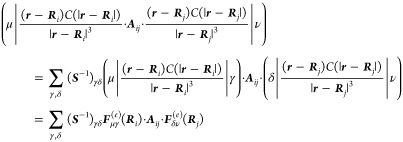
23

With these definitions, the matrix elements of the polarization Hamiltonian in eq 8 consist of the following parts:

- Two-electron part

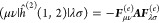
24The two-electron polarization integrals have the same symmetry under the permutation of orbital indices as the electron repulsion integrals
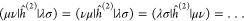
25These will not be formed directly, but rather, we make use of their tensor factorizable nature in terms of one-electron integrals, as discussed below. The effective polarizability supermatrix, ***A***, is constructed by the inversion of eq 5 using LU decomposition. While this carries a computational cost that scales as *N*_pol_^3^, the inversion needs only to be carried out once for a given molecular geometry before the electronic structure calculation is started. As a result, it carries a low prefactor and does not dominate the observed scaling of our method, at least for the range of *N*_pol_ we explored. For very large numbers of polarizable sites, the inversion to form ***A*** could dominate the overall computational scaling. In the future, we will explore iterative inversion approaches.

- One-electron part

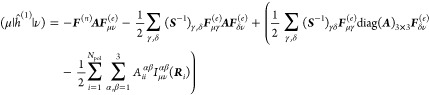
26These one-electron-polarization contributions are simply added to the core Hamiltonian. In the second term of eq 26, the same-site contributions (*i* = *j*) are treated with the resolution-of-identity trick discussed above.^49^ The last term in brackets removes these and replaces them by the exact integrals for *i* = *j*. diag(***A***)_3×3_ contains only the diagonal 3 × 3 blocks of ***A***. If one wishes to treat all integrals on the same footing and with the resolution of identity, the term in parentheses may be omitted.

Since the number of polarizable MM atoms, *N*_pol_, may be quite large, it is preferable to evaluate the contractions in such a way that the exponent *k* of the scaling relation  is as low as possible. First, the two tensors
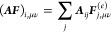
27
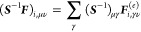
28are calculated. The first operation scales quadratically in *N*_pol_ (two loops are needed, one over *i*, the other over *j*), while the second one scales linearly. Then, the resolution-of-identity part of the core Hamiltonian is constructed as
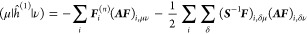
29which again scales linearly in *N*_pol_. The term in parentheses in eq 26 already has the desired linear scaling. The only operation that does not scale linearly is the construction of (***AF***)_*i*,μν_, which scales quadratically with *N*_pol_. Future work will explore how to reduce this to linear scaling.

- And finally the zero-electron part

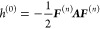
30which is added to the (classical) nuclear–nuclear repulsion and any of the nonpolarizable MM force-field terms. Note: this term includes the MM polarization energy, which therefore should not be evaluated separately outside IERDRF, to avoid double counting.

The construction of the Coulomb and exchange parts of the Fock operator requires efficient algorithms to evaluate contractions of electron repulsion integrals (μν|γδ) with molecular orbital coefficients *C*_γ*k*_ (*k* is the index of an occupied molecular orbital). The analogous sums for the two-electron part of the polarization Hamiltonian factorize and can thus be efficiently calculated, provided that the number of polarizable sites is not too large.

For Hartree–Fock theory, the IEDRF correction terms for Coulomb and exchange operators are
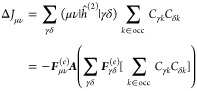
31and
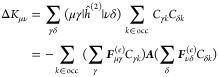
32respectively.

Efficient implementations of TD-DFT, CIS or CASSCF theories are formulated in terms of generalized Coulomb- and exchange-like matrices constructed from different one-particle density matrices, ***D***,^50^ which do not have the simple form *D*_μν_ = ∑_*k*∈occ_*C*_μ*k*_*C*_ν*k*_ of a closed shell Slater determinant. Equations 31 and 32 then have to be modified as
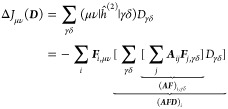
33and
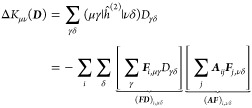
34

The sums over *j*, γδ (γ and *j*) in the construction of *J* (*K*) have the form of matrix–matrix multiplications, which can make use of the parallelism of GPUs. In the next step, each matrix element (μ, ν) of the Coulomb and exchange matrices is computed by one thread on the GPU.

As noted earlier, the construction of (***AF***)_*i*,γδ_ carries a computational cost that scales as *N*_pol_^2^, and this term appears in both the *J* and *K* builds. We avoid the construction of this term dominating the scaling of the method by noting that it does not depend on any density matrix; therefore, we precalculate it with a storage cost that matches the field integrals. In the current implementation, we do not take advantage of sparsity in the AO representation of the density matrix, *D*_γδ_, or the field integrals, ***F***_*i*,μγ_. As a result, for small to moderate numbers of polarizable sites, the scaling of the method is dominated by the contraction of the two inner terms in the exchange matrix of eq 34: (***FD***)_*i*,μδ_ with (***AF***)_*i*,νδ_. This contraction has a scaling of *N*_pol_ × *N*_AO_^3^, and while linear scaling in the number of polarizable sites (confirmed below), comes with a relatively high prefactor due to the cubic scaling with QM size. One also expects that *N*_pol_ itself will scale with the solvent-accessible surface area of the solute, which for linear molecules scales as *N*_QM_ and for globular molecules scales as *N*_QM_^2/3^. This will further increase the scaling of the method by up to an additional power of *N*_AO_. Future work will address this bottleneck by taking better advantage of the sparsity of the matrices in the AO representation.

Polarization is treated on the same footing for all electronic states. Nevertheless, the induced polarization and polarization energies are state-dependent quantities. Given the density matrix *D*_μν_ for an electronic state with the total charge density ρ, we can compute the mean-field (expectation) electric field generated at the polarizable sites as
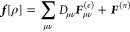
35The induced dipoles are related to the fields by the effective polarizability
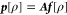
36and the mean-field polarization energy for the particular state is then
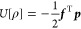
37

### 2.3 Analytical Gradients

Since the DRF approach directly modifies the one- and two-electron integrals of the QM Hamiltonian, analytical gradients are relatively straightforward to derive, adding modified integral derivative terms to the gradient expressions of the underlying electronic structure theory. For example, QM/MM-IEDRF embedding modifies the gradients of RHF^51^ to
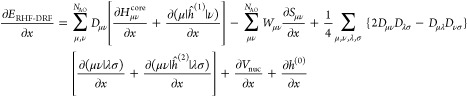
38where *x* represents any external parameter, which in our case could be the coordinates of the nuclei, the point charges, or the polarizable sites. The gradient depends on the following quantities
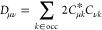
39is the RHF density matrix and
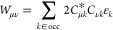
40is the “energy-weighted” density matrix, both in the atomic orbital basis. Here, *C*_μ*k*_ are the coefficients of occupied molecular orbital *k* with orbital energy ε_*k*_. (μν|λσ) are the two-electron repulsion integrals, *H*_μν_^core^ and *S*_μν_ are the core Hamiltonian and the overlap matrix, respectively, and *V*_nuc_ is the nuclear–nuclear repulsion energy. All of the integral derivatives arising from IEDRF have analytical expressions, although they are rather lengthy and therefore discussed in the Supporting Information.

In order to limit the memory footprint of the algorithm, it is convenient to avoid the storage of large arrays with integral derivatives. Instead, gradients of matrix elements are immediately contracted with a density matrix. These contracted gradients are thus functions of up to two density matrices, labeled ***D***^(1)^ and ***D***^(2)^. Although the expressions for RHF gradients involve the same density matrix, ***D***^(1)^ = ***D***^(2)^, keeping separate labels in the equations below allows for immediate generality to contractions that arise in the analytical gradients of other electronic structures, including CIS, TD-DFT, and CASSCF,^44^ where the density matrices can be different and are not necessarily symmetric.

In the same way that IEDRF introduces corrections to Coulomb and exchange operators (eqs 31 and 32), corrections to gradients of Coulomb and exchange operators can be formed
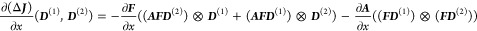
41where the argument of the function ∂***F***/∂*x* contains Kronecker products between a vector of size *N*_pol_ and a density matrix of dimensions *N*_AO_ × *N*_AO_, e.g.
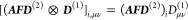
42and the function ∂***F***/∂*x* contains contractions of the derivative polarization integrals, ∂***F***^(*e*)^/∂*x*, with a supertensor ***E*** of dimensions 3 × *N*_pol_ × *N*_AO_ × *N*_AO_, computed in an integral-direct fashion
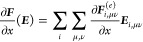
43The polarization integral derivatives are evaluated inside the loop over *i*, μ and ν. They are directly multiplied with the corresponding matrix element of the density matrix and are added to the gradient ∂***F***/∂*x*. Similarly, we define the contraction of a 3 × 3 × *N*_pol_ × *N*_pol_ supermatrix ***U*** with the gradient of the effective polarizability
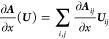
44

The corrections to gradients of the exchange operator follow in a similar fashion
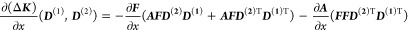
45where the superscript T indicates a matrix transpose. A full derivation of these expressions is provided in the Supporting Information.

## 3 Results

### 3.1 Solvatochromism of Intramolecular Charge-Transfer States

**Figure 1:**
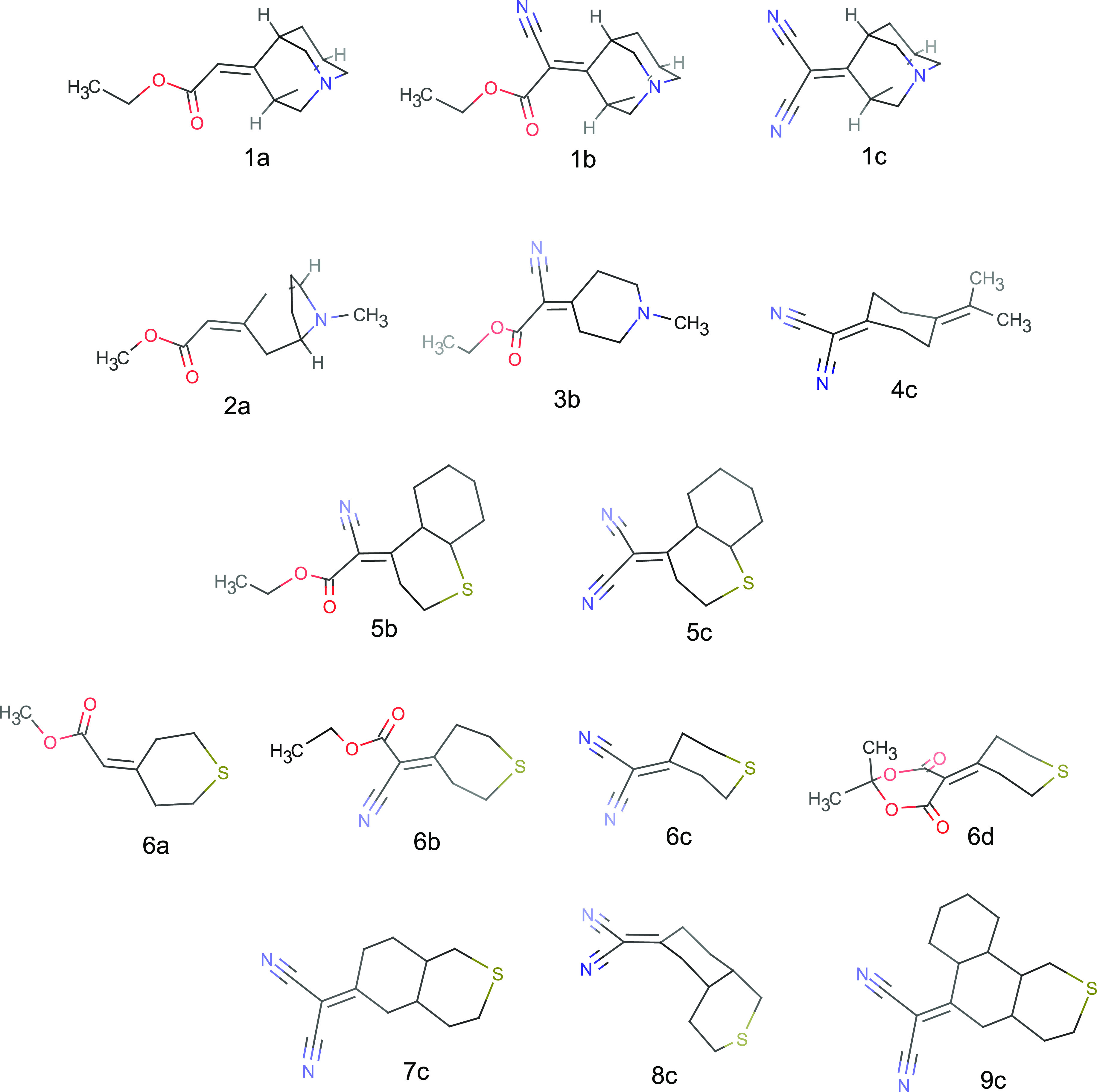
Chemical structures of the selected bichromophoric dyes from Pasman et al.^52^

**Figure 2:**
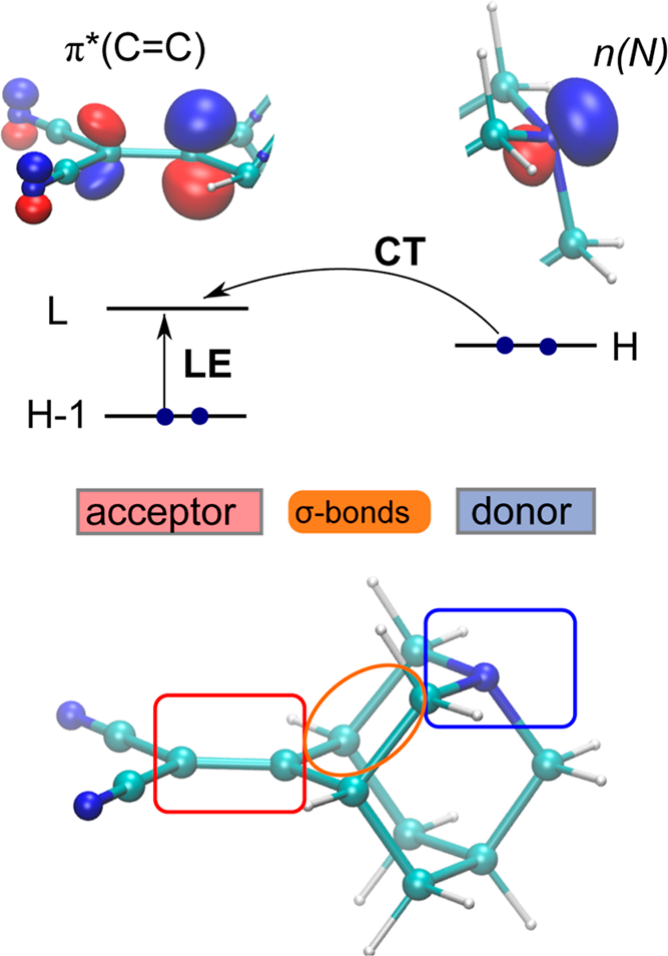
Frontier orbitals of the dye **1c**.

Pasman et al. synthesized a series of bichromophoric dyes to study intramolecular charge transfer.^52^ The charge-transfer states are visible in the absorption spectra and thus provide experimental reference energies against which our calculations can be benchmarked. The dyes consist of an acceptor and donor region separated by several σ bonds. Chemical structures of the dyes are shown in Figure 1. An electron is donated by the lone electron pair of a tertiary amine (systems 1, 2, 3) or a sulfur atom (systems 5, 6, 7, 8, and 9) or an electron-rich double bond (system 4) on one end of the molecule. The acceptor is the π* orbital of a double bond substituted with electron-withdrawing groups such as cyano or ester groups on the other end. Electron donor and acceptor moieties are separated by one or two cyclohexane rings. The frontier orbitals of one of the dyes are listed in Figure 2. The orbitals are localized on the donor and acceptor fragments and give rise to a local excitation (LE) of the C=C bond on the acceptor and a lower-lying charge transfer (CT) state, where an electron is transferred from the lone pair to the π* orbital of the C=C bond. In this idealized picture, the long-range CT state would be dark; however, in reality, there is significant mixing between the LE and CT states due to through-bond coupling, so that the CT transition borrows intensity from the LE transition and both states have large permanent dipole moments. This allows both the LE and CT states to be identified in a UV/vis absorption spectrum.

#### 3.1.1 Optimal Tuning in the Gas Phase and in Solution

Since charge-transfer states are notoriously difficult to model with local and hybrid density functionals, an optimally tuned range-separated hybrid is employed, which has been shown to give excellent fundamental gaps of atoms and molecules in the gas phase.^53^ We determine the optimal range-separation parameter separately for each dye in a vacuum and in solution.

The geometries of all dyes were optimized in the gas phase at the ωPBEh^54^/def2-SVP level of theory using the default range-separation parameter ω = 0.2 bohr^–1^ and the default value of *C*_HF_ = 0.2 for the portion of exact Hartree–Fock exchange at full range. For dyes with cyclohexane rings, the most stable chair conformation was chosen. At the double bond of the acceptor, we selected the cis/trans isomer that afforded the least steric hindrance. The range-separation parameter was tuned^54,55^ separately for each dye in the gas phase and in solution (see Supporting Information Section 3). For tuning the functional in solution with the direct reaction field, snapshots of dyes embedded in *n*-hexane were generated, as described later.

Depending on the dye, the optimal vacuum range-separation parameter varies from ω_opt_^vacuum^ = 0.17 bohr^–1^ (for **1b**) to 0.22 bohr^–1^ (for **6a**). The values obtained from tuning in *n*-hexane with the QM/MM embedding scheme are approximately 5% larger than in the gas phase, and with QM/MM-IEDRF they are approximately 10% larger ranging from ω_opt_^QM/MM–IEDRF^ = 0.20 bohr^–1^ (for **1b**) to 0.24 bohr^–1^ (for **6c**). This finding is in contrast to state-specific polarization, in which the optimal value of omega is found to approach 0 as the dielectric constant of the environment increases.^56^ A similar issue was noted when applying optimal tuning to condensed-phase systems and motivated the development of screened range-separated hybrid functionals that incorporate a fraction of long-range exact exchange equal to 1/ϵ.^57−59^ Since IEDRF already includes the effects of screening at the Hamiltonian level on both one-particle and many-body state energies, the fraction of exact exchange at long-range can be set equal to 1.0, while still yielding reasonable optimal omega values. This is very promising for quantitative prediction of both LE and CT states, which we demonstrate below. The optimal values for all dyes are listed in the Supporting Information.

#### 3.1.2 Solution-Phase Absorption Spectra: *n*-Hexane

We now describe the procedure for solvating the chromophores and sampling snapshots of nuclear geometries along a classical molecular dynamics trajectory. The influence of electronic polarization on the absorption spectra is then estimated by calculating vertical excitation energies on those snapshots with and without the direct reaction field.

##### 3.1.2.1 System Setup

GAFF parameters^60^ were assigned for both the chromophores and the *n*-hexane solvent molecules using *antechamber*(61) and *leap* from the AMBER 2018 package.^62^ The chromophore was packed into a ball of 5000 solvent molecules with the help of the Packmol program.^63^ The radius of the sphere was determined from the experimental density at room temperature. An octahedral unit cell was carved out of the solvent ball to allow for simulations under periodic boundary conditions.

All MM optimizations and molecular dynamics simulations were performed with *pmemd.cuda* or *sander* from the AMBER 2018 package.^62^ A nonbonded cutoff of 8 Å was used in all simulations, with the particle-mesh Ewald summation^64^ used to handle long-range electrostatic interactions. Bonds to hydrogen atoms were constrained using the SHAKE algorithm.^65^

##### 3.1.2.2 Heating and Equilibration

The system was relaxed in two steps, first by optimizing only the solvent molecules while restraining the solute atoms with a harmonic force constant of 100 kcal/(mol·Å^2^), followed by relaxing the whole system for 10,000 optimization steps. The system was gradually heated up from 0 to 300 K over 100 ps. The system was then equilibrated for 1 ns with a 1 fs time step in the *NVT* ensemble at 300 K with a Langevin thermostat and a collision frequency of 1.0 ps^–1^. This was followed by a second equilibration lasting also 1 ns in the *NPT* ensemble at a temperature of 300 K and a pressure of 1 bar using the Berendsen barostat^66^ with a pressure relaxation time of 1.0 ps.

##### 3.1.2.3 Production

Starting with the equilibrated coordinates and velocities, a single trajectory was run for 10 ns in the *NPT* ensemble. Snapshots were taken every nanosecond, yielding 10 uncorrelated configurations.

##### 3.1.2.4 Validation

At the end of the production run, some basic consistency checks were performed: (a) the total energy and temperature were observed to be stable; (b) the average density was computed. This is a sensitive test for the nonbonded interactions between the solvent molecules. In all cases, the density was within 5% of the experimental value of 0.6606 kg/m^3^.^67^

##### 3.1.2.5 Postprocessing of Snapshots

For the QM/MM-IEDRF calculations, atomic dipole polarizabilities from Applequist’s model^29^ were assigned to the MM atoms. In order to reduce the computational expense, polarizabilities of solvent molecules farther than 5 Å away from the QM region were coarse-grained, as explained below in Section 3.4. MM atoms were also equipped with effective core potentials to avoid the electron spill-out problem.^68^ In the QM/MM-IEDRF calculations, the small MM point charges on the *n*-hexane molecules were also included in the electrostatic potential. It should be noted that the partial charges on *n*-hexane’s atoms are very small (the terminal hydrogen charges are 0.0327*e*), consistent with the apolar nature of this solvent. As a result, electrostatic interactions are negligible, and the solvatochromism is dominated by the polarization Hamiltonian.

Excitation energies are very sensitive to small changes in the bond lengths. Since the GAFF force field was not sufficiently reliable at maintaining the equilibrium structures of the dye molecules, the snapshots extracted from the MM trajectory contained chromophores with slightly wrong bond lengths. In order to fix this, the solvated systems were optimized for 100 steps at the QM/MM level of theory (and separately at the QM/MM-IEDRF level) using the ωPBEh functional (with default parameters) and the def2-SVP basis set. Outer solvent molecules with coarse-grained polarizabilities were frozen during the optimization. Since TeraChem does not yet support periodic boundary conditions, the octahedral unit cell was used as a solvent “droplet”. For the gas-phase reference calculations, the chromophores were taken out of the solvent and optimized locally with the same functional and basis set.

### 3.2 Vertical Absorption Spectra with TD-DFT

**Figure 3:**
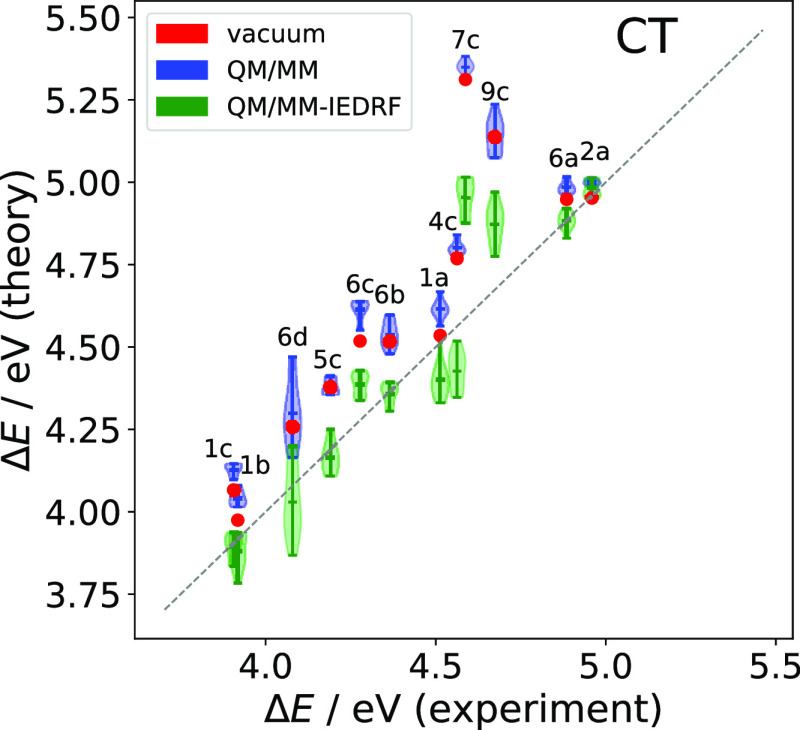
Correlation between experimental absorption band maxima (experiment) of the charge transfer state and the lowest vertical TD-ω_opt_PBEh/aug-cc-pVDZ (theory) excitation energy with different embedding schemes: isolated chromophore in gas phase (vacuum, red circles), electrostatic (QM/MM, blue violin plots) and electrostatic + polarizable embedding (QM/MM-IEDRF, green violin plots). Violin plots indicate the distribution of energies among the 10 snapshots. The diagonal dashed line indicates a perfect correlation.

For each of the locally optimized snapshots, the lowest few excited states were obtained with linear-response TD-ω_opt_PBEh/aug-cc-pVDZ using the different embedding schemes. For consistency, the same embedding scheme was chosen as for geometry optimization. That is to say, the absorption spectrum with electrostatic embedding (QM/MM) was calculated at the QM/MM optimized geometry and the spectrum with electrostatic and polarizable embedding (QM/MM-IEDRF) at the corresponding QM/MM-IEDRF optimized one. Figure 3 shows a correlation plot between the experimental CT energies and the TD-DFT predictions with different embedding schemes.

Overall, the trends across most of the molecular structures are already reasonably well captured by the gas-phase TD-DFT calculations (red circles), taking into account that we are neglecting the vibronic structure and are comparing vertical excitation energies with absorption band maxima. However, some important differences between the theoretical gas- and solution-phase experimental results are noted. First, the dyes **3b** and **8c** show a low-lying CT state in our calculations not seen in experiment; however, their oscillator strengths are ≤0.01, which is much smaller than the oscillator strengths of the LE states (≥0.4). Therefore, these states are likely not identifiable in experimental UV/vis spectra. As for the dye **5b**, we suspect there to be a typo in Table 1 of ref (52), since **5b** and **6c** are listed with exactly the same CT energies, although their different structures and our TD-DFT calculations suggest them to be different. We therefore leave **3b**, **5b,** and **8c** out of our analysis. Second, with the exception of dyes **1a**, **1b**, **2a,** and **6a**, the gas-phase calculations overestimate the CT energies by 0.2 eV or more. In particular, the chromophores **7c** and **9c** stick out with differences between the theoretical gas-phase and experimental CT energies of 0.7 and 0.5 eV, respectively. This makes perfect sense, if one looks at their molecular structures in Figure 1: In both **7c** and **9c** the donor and acceptor moieties are separated by two cyclohexane rings, so that the electron and hole are kept far apart. Such long-range CT states are very sensitive to the dielectric environment. This motivates an atomistic representation of the liquid environment, which we turn to next.

In the QM/MM calculations (blue violin plots), the electrostatic interaction with the (fixed) point charges and Pauli repulsion is added. Since *n*-hexane is apolar, the MM charges are small so that no large effect is expected. Since the range-separation parameters ω_opt_^QM/MM^ are approximately 5% higher than in the gas phase, presumably due to the QM-MM Pauli repulsion, the increased exact exchange at medium range slightly shifts the excitation energies up. Indeed, for all chromophores, the means of the QM/MM excitation energies are higher than their respective gas phase values, worsening the agreement with experiment, which we attribute to the missing polarization interactions in electrostatic-embedded QM/MM, which especially should lower the energies of the CT states from the gas-phase to solution. In addition, dispersion interactions with the apolar *n*-hexane molecules, missing in the gas phase, should lower the energies of all excited states, since excited states usually are more diffuse and polarizable than the ground state. On the other hand, the Pauli repulsion tends to compress the wave functions of diffuse states in the solvent cage and can raise the excitation energy again. The different configurations in each snapshot lead to an inhomogeneous broadening of >0.1 eV. This broadening is largely due to the conformational flexibility of the dyes and does not depend much on the embedding scheme. **6d**, which has the largest spread of almost 0.5 eV, occurs in two stable conformations which differ in the orientation of the isopropyl group on the acceptor moiety.

Now, turning on polarization interactions in the QM/MM-IEDRF calculations (green violin plots), the presence of the solvent enters in the form of the DRF Hamiltonian, which modifies both the one- and two-electron integrals and effectively screens (or “renormalizes”) the Coulomb interaction. The CT energies are lowered, bringing them into closer agreement with experiment. Furthermore, the magnitudes of the solvatochromic shifts are consistent with the range of the charge transfer: The long-range CT states in dyes **7c** and **9c** are lowered most, as expected. This supports a view that the solvatochromism of these dyes is dominated by differential induced polarization of the solvent between the ground and excited states. For dyes **1a** and **4c**, the CT energies are slightly underestimated by approximately 0.1 eV, which is, anyway, within the expected accuracy of TD-DFT. Nevertheless, the significant improvement in CT excitation energies observed going from electrostatic-embedded QM/MM to QM/MM-IEDRF is highly encouraging.

### 3.3 QM/MM-IEDRF Optimization of Conical Intersections

At the Franck–Condon point, the CT and LE states are mixed, so that both the S_1_ and S_2_ states acquire some oscillator strength. Emission happens from the lowest excited state, which is strongly affected by the polarity of the solvent. The transition between the absorbing and the emitting states is mediated by a conical intersection. The mixing coefficients between the CT and LE states can change on the path to the conical intersection, so that its topography and energetic location could be sensitive to solvent polarization.

With IEDRF, it is possible to locate conical intersections reliably between states of different polarity in solution since the polarization Hamiltonian does not depend on a particular state of interest. To demonstrate this, we take the dye with the largest solvatochromic shift, **7c**, and search for the minimal energy conical intersection (MECI) between the S_2_ and S_1_ states in *n*-hexane with the SA-3-CASSCF(4e,3o)/def2-SVP method: The minimal complete active space contains four electrons in three orbitals, comprising the highest occupied orbital of the donor and the π and π* orbitals of the double bond in the acceptor. The lowest three singlet states (ground, CT and LE) are included in the state averaging. The coordinates of the solvent molecules with coarse-grained polarizabilities are frozen during the optimization, so that only the chromophore and the inner solvent shell are allowed to move.

**Figure 4:**
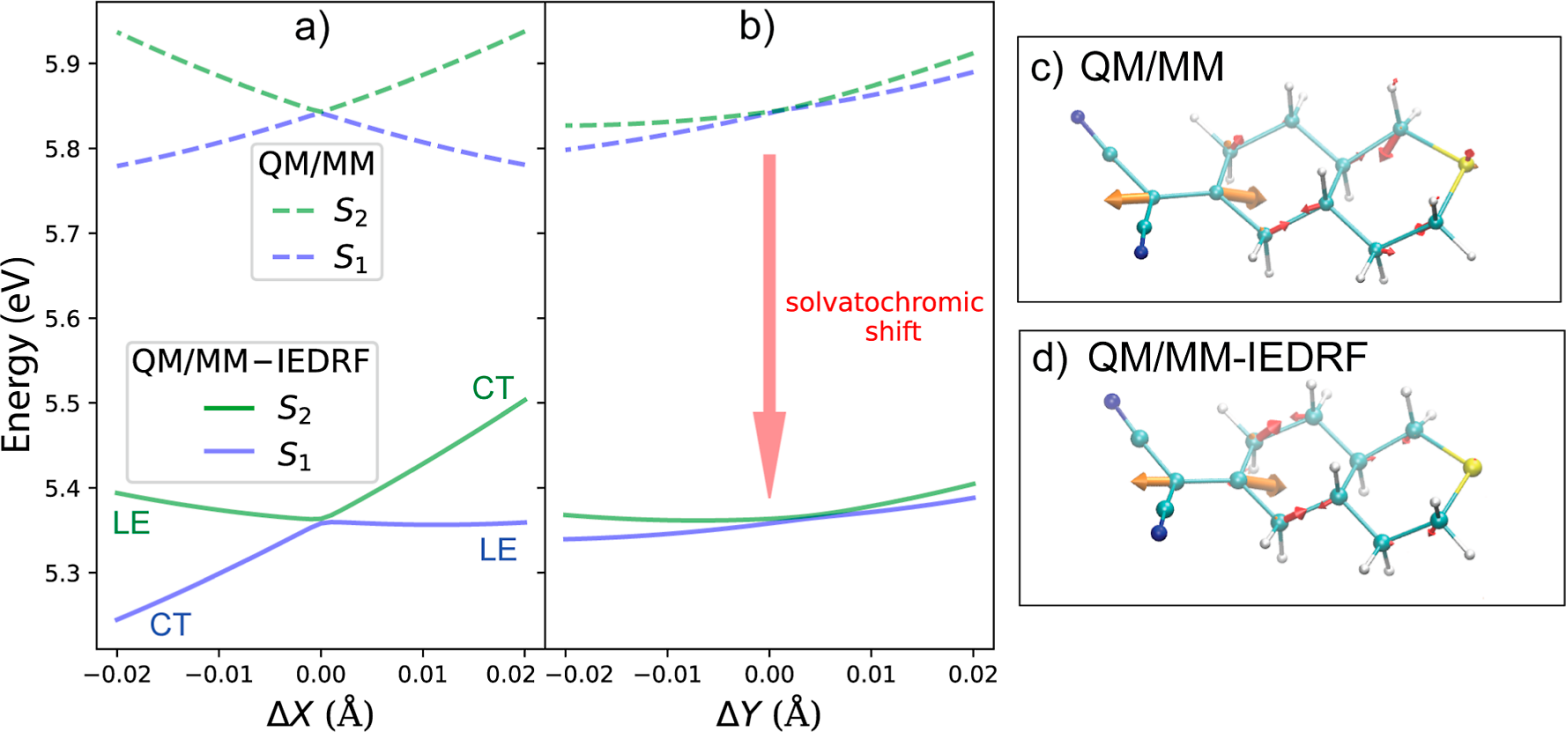
S_2_/S_1_ conical-intersection branching space for a representative snapshot of **7c** in *n*-hexane. Panels (a,b) show potential energies along the nonadiabatic coupling vector (Δ*X*) and gradient difference vectors (Δ*Y*) respectively. Dashed lines: electrostatic and Pauli embedding (QM/MM). Solid lines: electrostatic, Pauli and polarizable embedding (QM/MM-IEDRF). Panels (c,d) show the QM/MM MECI and QM/MM-IEDRF MDCI geometries respectively (see main text for details). The orange and red arrows correspond to Δ*X* and Δ*Y,* respectively.

Starting from the Franck–Condon point of a representative snapshot, the S_2_/S_1_ MECI was optimized at the QM/MM level with the gradient projection method using DL-FIND.^69^ Then, the minimal distance conical intersection (MDCI) to the MECI was reoptimized at the QM/MM-IEDRF level. Figure 4c,d shows the optimized MECI/MDCI geometries together with the vectors that lift the degeneracy of the branching space for a representative snapshot. For both embedding schemes, the nonadiabatic coupling vector and the gradient difference vector are essentially fully localized on the chromophore: the components of the vectors on the solvent atoms are too small be visualized. The MECI is characterized by an elongation of the double bond, a pyramidalization of the dicyanovinyl group and an increased puckering of the cyclohexane ring at the sulfur atom, much like in the gas phase.

Despite the CI geometries and the branching space vectors being very similar for QM/MM and QM/MM-IEDRF (by construction, due to the latter being optimized to an MDCI biased to the former’s MECI structure), panels (a,b) of Figure 4 show noticeable differences in the potential energies in the two-dimensional branching space where the degeneracy between the two states is lifted. First, when the induced polarization is taken into account with IEDRF, the conical intersection is lowered by ∼0.5 eV. This can be understood by the mixed LE/CT character in both S_1_ and S_2_ at the intersection, such that they are both stabilized by solvent polarization. In addition to this overall stabilization, the topography of the intersection is affected by polarization: at the QM/MM level, the intersection is peaked in the nonadiabatic coupling vector direction but becomes somewhat sloped at the QM/MM-IEDRF level. This can be understood by S_1_ having CT character for geometries displaced in the negative Δ*X* direction, leading to it being more stabilized by solvent polarization than S_2_. On the other hand, both states retain mixed LE/CT character for displacements along the gradient difference vector, and therefore the shape of the potential energy surfaces in this direction is not significantly changed by the addition of polarizable embedding. Overall, these findings highlight the importance of including a description of induced polarization in the environment when exploring conical intersections involving CT states.

### 3.4 Comparison with Full-System Calculations

To verify that our QM/MM-IEDRF method faithfully captures solvent–solute interactions, we compared its predictions with those of full-system DFT calculations, where a large number of solvent molecules are included in the QM region. Since TD-DFT becomes rather expensive for large systems, the energy of the lowest triplet excitation is instead targeted, which can be obtained as the difference of two self-consistent field calculations.

The lowest singlet state S_0_ and triplet state T_1_ are calculated by using the (unrestricted) ωPBEh/def2-SVP method with the default range separation parameter of ω = 0.2 bohr^–1^. The QM/MM calculations are performed with electrostatic embedding + Pauli repulsion, which yield values close to gas-phase results since *n*-hexane has very small partial charges. The QM/MM-IEDRF calculations include these same interactions in addition to the polarizable embedding. In the full-system calculations, any solvent molecule that intersects a shell of 5 Å around the chromophore is included in the QM region. The chromophore and solvent geometries are exactly the same in the embedding schemes and the full-system calculations.

**Figure 5:**
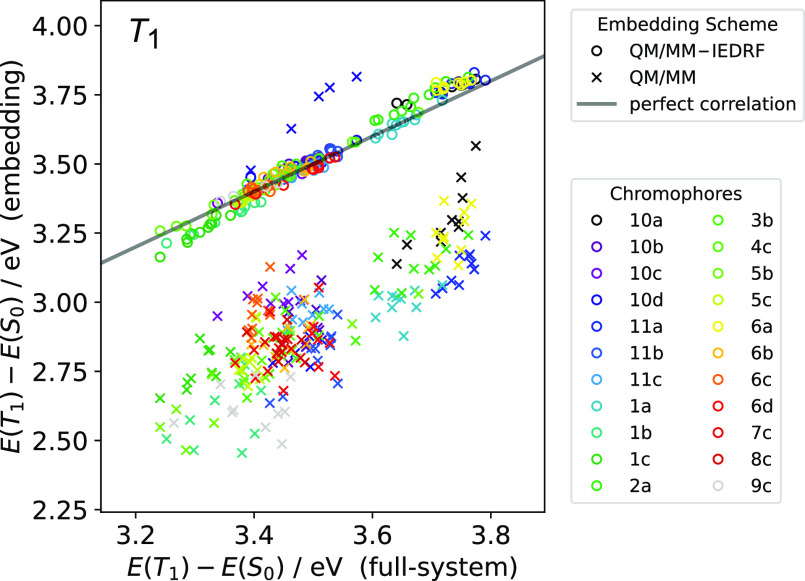
Correlations between embedding- and full-system calculations of the lowest singlet–triplet excitation energy, for all snapshots and dyes. The gray line indicates a perfect correlation. For some snapshots the SCF cycle for the triplet state did not converge; those snapshots are not shown.

Figure 5 shows that QM/MM-IEDRF (circles) agrees essentially quantitatively with the full-system calculations, while neglecting the electronic polarization in the solvent (QM/MM, x marks) leads to an underestimation of the lowest triplet excitation by up to almost 1 eV. The root-mean-square deviation from the full system reference energies is 0.03 eV for QM/MM-IEDRF and 0.6 eV for QM/MM. The lowest triplet T_1_ is predominantly a local excitation on the C=C acceptor group. Since the singlet ground state has a slightly larger dipole moment than T_1_, a polarizable environment actually increases the gap between S_0_ and T_1_. There is also a higher excited triplet state with long-range charge-transfer from the donor to the acceptor. For instance, for one snapshot of **7c**, the SCF accidentally converged to the triplet with CT from the sulfur lone pair to the C=C acceptor, which is much higher in energy than the first triplet state. However, it proved difficult to converge to this state with the Δ-SCF approach. The QM/MM outliers above the diagonal in Figure 5 are due to convergence problems.

### 3.5 Convergence and Computational Cost

**Figure 6:**
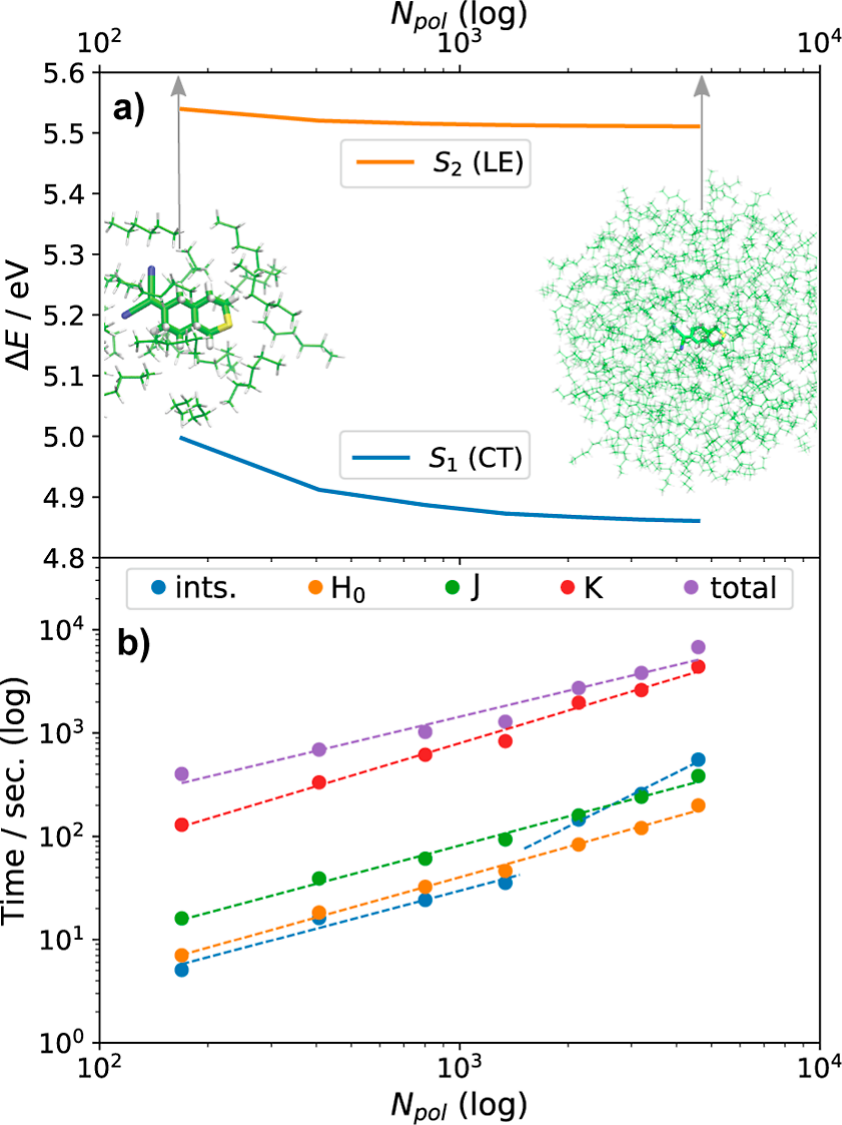
TD-ωPBEh/6-31g* calculation for the lowest 8 excited singlet states of the dye **7c** (QM) in *n*-hexane (MM): (a) Convergence of excitation energies with the size of the solvent shell. (b) Timings for preparing integrals (ints.), core Hamiltonian (*H*_0_), and J- and K-builds on 2 × NVIDIA GeForce GTX 1080 Ti GPUs.

We now focus on the convergence and scaling of the computational cost with the system size. The convergence of singlet vertical excitation energies of a solute (treated at the time-dependent density functional theory level) with a number of solvent molecules (treated at the IEDRF level) is explored in Figure 6 for dye **7c** in *n*-hexane. As expected, the LE state converges rapidly with the size of the MM region, reaching its plateau value at a radius of ∼8 Å, while the CT state requires a rather larger radius of ∼14 Å to reach convergence. Polarization is an inherently long-range phenomenon; therefore, these findings are not surprising.

As discussed in Section 2, the current formulation of QM/MM-IEDRF has a computational bottleneck in the exchange operator that is linear scaling with the size of the QM system, albeit with a rather large prefactor. This is demonstrated in Figure 6b which confirms linear scaling: The size of the solvent shell is measured by *N*_pol_, which is the number of polarizable MM atoms. The timings for preparing integrals (ints), construction of core Hamiltonian (*H*_0_), and J- and K-builds on 2 × NVIDIA GeForce GTX 1080 Ti GPUs are shown on a log–log scale. For large solvent shells, most of the time is spent on IEDRF corrections to the Hamiltonian, in particular the K-build. Scaling exponents were determined from fits to *CN*_pol_^*m*^ (shown as dashed lines): core Hamiltonian *H*_0_ ∝ *N*_pol_^0.98^; J-build ∝ *N*_pol_^0.93^; K-build ∝ *N*_pol_^1.05^; total ∝ *N*_pol_^0.83^. For *N*_pol_ < 1500, the preparation of constant intermediates is dominated by the evaluation of the polarization integrals, which scales linearly (∝*N*_pol_^0.92^), but for *N*_pol_ > 1500, constructing AF (eq 27) becomes the bottleneck with approximately quadratic scaling (∝*N*_pol_^1.74^).

The wall times of the current implementation are rather high (albeit much lower than a full-system TD-DFT calculation of the same system size) and preclude ab initio dynamics at present. Possible remedies have already been explored in the original formulation of DRF: at large distances, the atomistic nature and orientation of the solvent molecules do not matter, so that distant parts of the solvent can be replaced by a continuum.^32^ This suggests a multilayered partitioning of the system: an inner QM region is surrounded by a thin shell of explicit polarizable MM atoms, which in turn is enclosed by a polarizable continuum (see Figure 1 in ref (70)). Another simpler solution is to combine the atomic dipoles on a distant solvent molecule into a single polarizable site (with a possibly anisotropic molecular polarizability) placed at the molecular center. This does not change the scaling but reduces the number of polarizable sites by coarse-graining the reaction field far from the QM region.

**Figure 7:**
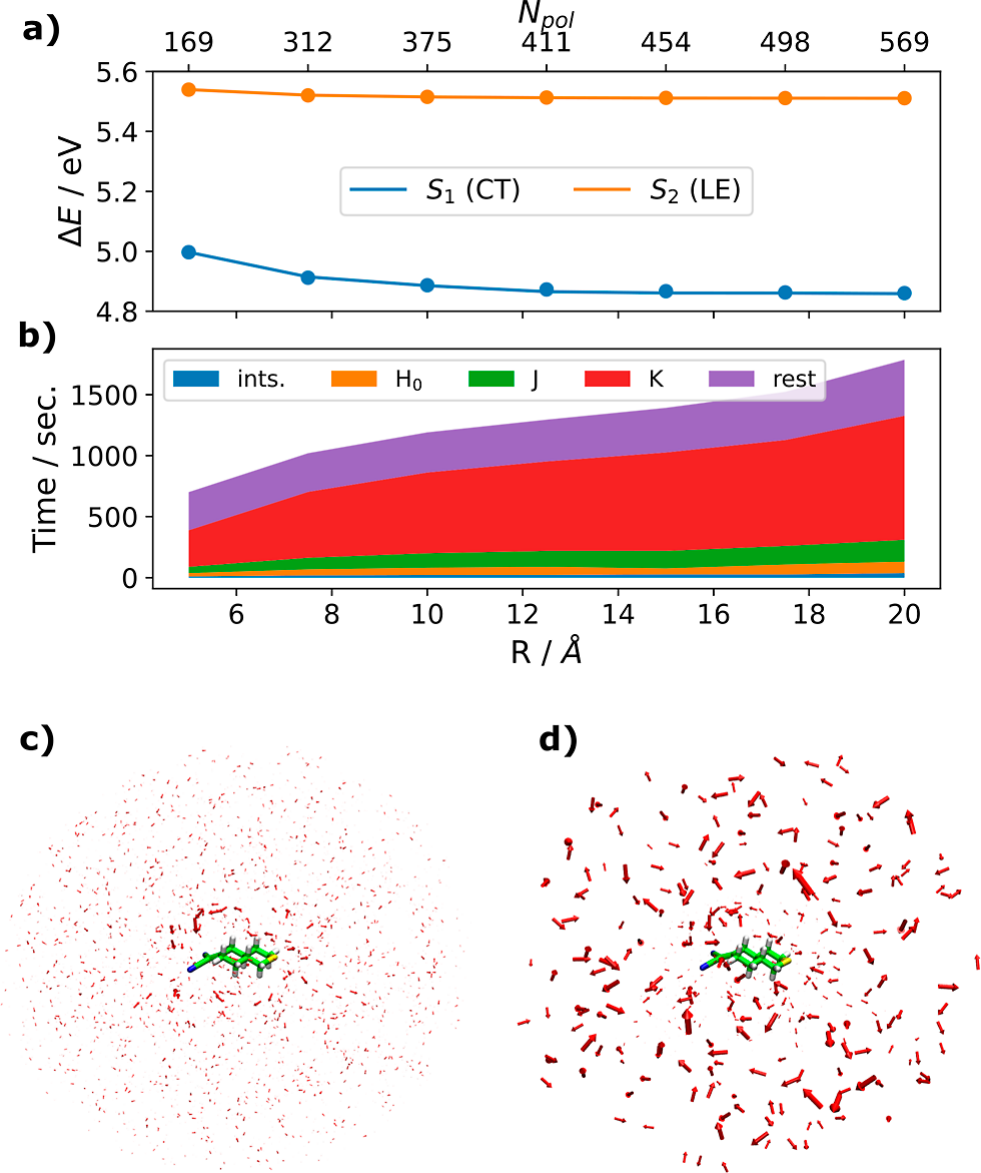
Same calculation as in Figure 6 with coarse graining. (a) Solid lines are the energies for a coarse-grained reaction field, dots mark energies for a fully atomistic reaction field; (b) timings; (c) and (d) induced dipole moments in the S_1_ (CT) state: (c) atom centered dipoles, (d) dipoles outside a sphere of radius 5 Å are combined into molecule-centered dipoles.

The time savings of such a coarse-graining approach are illustrated in Figure 7. The coarse graining is applied to solvent molecules if all of their constituent atoms are farther than 5 Å from any QM atom. Atom-centered dipoles are replaced by a single dipole, which is placed on the atom closest to the center of mass. The polarizability of the molecule-centered dipole is estimated according to Applequist’s and Thole’s dipole interaction model.^29,45^ To avoid dependence on the orientation and internal coordinates, the molecular polarizability tensor **α**_mol_ is diagonalized and the average of the eigenvalues is taken as the isotropic, scalar polarizability: . Figure 7 shows that grouping atomic dipoles on distant solvent molecules together hardly changes the energies but reduces the computation time significantly.

## 4 Justification for Combining DRF and DFT

Before concluding, we point out a fundamental open question about how to combine DRF with density functional theory. For large QM regions, time-dependent density functional theory is currently the most practical Ab initio electronic structure method. From a technical perspective, there is no obstacle to combining DFT (and TD-DFT) with QM/MM-IEDRF, by applying the DRF operators to density matrix elements of the noninteracting Kohn–Sham (KS) reference state. However, the theoretical justification for using the same exchange–correlation functionals as in a vacuum requires some further considerations. In particular, the first Hohenberg–Kohn Theorem (HK1) holds for external potentials that are one-electron operators.^71^ The DRF polarization Hamiltonian therefore does not qualify as an external potential. On the other hand, the DRF Hamiltonian modifies the electron–electron Coulomb interaction due to screening by the environment and since HK1 is not limited to any particular form of the electron–electron interaction, it guarantees that there is an appropriate density functional. However, the exact functional would be different for each solvent configuration and polarizable environment. To some extent, our use of optimally tuned range-separated hybrids in the IEDRF environment captures a difference in the exchange–correlation potential between the vacuum and condensed phase and ensures that the functional satisfies Janak’s theorem.^72^ The resulting tuned range-separation parameters are slightly higher than in vacuum. This procedure is seen to improve overall agreement with experiment: using the smaller vacuum values for ω_opt_ instead with DRF leads to an overestimation of the solvatochromic shifts.

From a pragmatic perspective, the observation that triplet excitation energies for a series of dye molecules computed with IEDRF embedding agree essentially quantitatively with full-system DFT calculations (Figure 5) gives us confidence that DRF can be reliably combined with DFT by using the KS noninteracting reference density matrix. This can be understood by noting that the solvent polarization, and therefore solvatochromic shift, should be dominated by the total electrostatic field arising from the real-system QM charge distribution, which is, by construction, reproduced by the KS reference state.

## 5 Conclusions

A polarizable embedding scheme for QM/MM simulations involving excited states has been presented, which is based on the direct reaction field method. In addition to having point charges, the MM atoms are equipped with point dipoles that interact with each other and react to the motion of the electrons in the QM region. The orientation of the solvent molecules is responsible for the static polarization of the solvent, which varies widely between polar and nonpolar solvents, while the induced dipoles capture the (infinite frequency) electronic polarization, which is rather similar for most solvents.

Different excited states of the QM part induce state-dependent polarization responses in the MM region. Nevertheless, the interaction between QM and MM regions is described by a single Hamiltonian that is the same for all electronic states. We improve upon the original method by evaluating the polarization integrals exactly, leading to the name IEDRF. Induction and dispersion are accounted for by the polarization Hamiltonian, while Pauli repulsion is mimicked by effective core potentials placed on the MM atoms. The fact that all excited states are eigenfunctions of the same Hamiltonian allows electronic state crossings of solvated molecules to be defined in a consistent manner. This point has been illustrated by optimizing the minimal energy crossing point of a solute dye for two excited states with different polarities in a solvent shell.

Our implementation of the direct reaction field exploits TeraChem’s formulation of quantum-chemistry methods in terms of a minimal set of basic kernel operations (Coulomb and exchange builds). The polarization Hamiltonian can be absorbed into the one- and two-electron integrals. After the core Hamiltonian and the Coulomb and exchange operators are modified, many quantum-chemistry methods work straight out of the box in combination with IEDRF embedding. Expressions were given for the additional core Hamiltonian, J- and K-parts, as well as all necessary derivatives needed for analytic gradients and couplings.

We illustrated the method for a series of bichromophoric dyes for which the absorption maxima of the lowest charge-transfer states in *n*-hexane were estimated from QM/MM-IEDRF calculations in combination with TD-DFT. Since *n*-hexane is a nonpolar solvent, the solvatochromic shifts relative to gas-phase TD-DFT values are mostly due to the electronic polarizability of the environment. QM/MM-IEDRF yields vertical S_0_–T_1_ excitation energies in essentially quantitative agreement (0.03 eV) with full-system QM calculations across the series of dyes. Optimization of a conical intersection in *n*-hexane reveals that inclusion of solvent polarization has an appreciable influence on the crossing, both stabilizing it by ∼0.5 eV relative to a QM/MM treatment and affecting its topography. These findings showcase the potential of QM/MM-IEDRF to be a highly accurate embedding method for photochemical and photobiological studies.

Although the computational cost of the current implementation precludes excited-state dynamics simulations with a large solvation shell, the method has a computational scaling and wall time much below those of full-system QM calculations of the same size. QM/MM-IEDRF is thus already practical for single-point vertical excitation energy calculations, geometry optimizations, and conical intersection searches, as demonstrated in this work. Future work will seek to lower the computational scaling with respect to the QM system size to enable excited-state dynamics simulations.

## Supporting Information Available

Background material on dipole field tensor, supervector notation, Thole damping, a detailed derivation of analytical gradients, protocol for optimal tuning of the range-separated hybrid functional, energies of the locally excited states, and discussion of dyes excluded from analysis (PDF)
Gas-phase structures of chromophores of Figure 3 (ZIP)
Representative snapshot with S_2_/S_1_ MECI/MDCI geometries of microsolvated **7c** from Figure 4 (ZIP)
