# Supplementary material for: Multistate, Polarizable QM/MM Embedding Scheme Based on the Direct Reaction Field Method: Solvatochromic Shifts, Analytical Gradients and Optimizations of Conical Intersections in Solution
Source: J Chem Theory Comput. 2024 Feb 8;20(5):2111–26. doi: 10.1021/acs.jctc.3c01018 (PMC10938509; doi:10.1021/acs.jctc.3c01018)
Supplement: Supplementary file 1 — ct3c01018_si_001.pdf [file ct3c01018_si_001.pdf]

Supporting Information for 'Multistate, polarizable QM/MM embedding scheme based on the direct reaction field method: solvatochromic shifts, analytic gradients and optimization of conical intersections in solution'

Alexander Humeniuk[*,†,‡] and William J. Glover[*,†,‡,¶,§]

†NYU Shanghai, 567 West Yangsi Road, Shanghai 200124, China

‡NYU-ECNU Center for Computational Chemistry at NYU Shanghai, 3663 Zhongshan Road North, Shanghai 200062, China

¶Shanghai Frontiers Science Center of Artificial Intelligence and Deep Learning, NYU Shanghai, 567 West Yangsi Road, Shanghai 200124, China

§Department of Chemistry, New York University, New York, New York 10003, USA

E-mail: alexander.humeniuk@gmail.com; william.glover@nyu.edu

1 Preliminaries

1.1 Dipole field tensor

The α-component of the electric field generated by a dipole $\boldsymbol{p}$ located at position $\boldsymbol{R}$ is given by

$$-\frac{\partial V_{\text{dipole}}}{\partial x_{\alpha}} = -\sum_{\beta} T_{\alpha\beta}(\boldsymbol{r} - \boldsymbol{R})\; p_{\beta}, \tag{SI-1}$$

with the symmetric 3×3 matrix (the dipole field tensor[1])

$$T_{\alpha\beta}(\boldsymbol{r}) = \frac{\delta_{\alpha\beta}}{r^3} - \frac{3}{r^5} x_\alpha x_\beta \qquad \text{with } \alpha, \beta = x, y, z. \tag{SI-2}$$

If there are N_{pol} dipoles it is convenient to combine the atomic vectors for the electric fields generated by the charge distribution, $\boldsymbol{f}_i[\rho]$, and the induced dipoles, $\boldsymbol{p}_i$, into super vectors of length $3N_{\text{pol}}$,

$$\boldsymbol{f} = (\boldsymbol{f}_1 \dots \boldsymbol{f}_i \dots \boldsymbol{f}_{N_{\text{pol}}})^T \tag{SI-3}$$

$$\boldsymbol{p} = (\boldsymbol{p}_1 \dots \boldsymbol{p}_i \dots \boldsymbol{p}_{N_{\text{pol}}})^T, \tag{SI-4}$$

and form the diagonal $(3N_{\text{pol}}) \times (3N_{\text{pol}})$ matrix

$$\boldsymbol{\alpha} = \text{diag}\left(\alpha_1 \mathbb{1}_{3x3}, \dots, \alpha_i \mathbb{1}_{3x3}, \dots, \alpha_{N_{\text{pol}}} \mathbb{1}_{3x3}\right), \tag{SI-5}$$

which contains the atomic polarizabilities on its diagonal, as well as the symmetric $(3N_{\text{pol}}) \times (3N_{\text{pol}})$ matrix

$$\boldsymbol{T} = \begin{pmatrix} 0 & \boldsymbol{T}^{(12)} & \dots & \dots & \boldsymbol{T}^{(1,N_{\text{pol}})} \\ \boldsymbol{T}^{(12)} & 0 & \dots & \dots & \dots \\ \vdots & \vdots & \ddots & \boldsymbol{T}^{(ij)} & \dots \\ \vdots & \vdots & \boldsymbol{T}^{(ij)} & \ddots & \boldsymbol{T}^{(N_{\text{pol}}-1,N_{\text{pol}})} \\ \boldsymbol{T}^{(1,N_{\text{pol}})} & \vdots & \vdots & \boldsymbol{T}^{(N_{\text{pol}}-1,N_{\text{pol}})} & 0 \end{pmatrix}, \tag{SI-6}$$

which has the dipole polarization tensors $\boldsymbol{T}^{(ij)} \in \mathbb{R}^{3\times3}$ for pairs of dipoles $\boldsymbol{p}_i$ and $\boldsymbol{p}_j$ in the off-diagonal positions.

With these abbreviations the polarization energy becomes

$$U = -\boldsymbol{p}^T \cdot \boldsymbol{f} + \frac{1}{2}\boldsymbol{p}^T \boldsymbol{\alpha}^{-1} \boldsymbol{p} + \frac{1}{2}\boldsymbol{p}^T \boldsymbol{T} \boldsymbol{p} \tag{SI-7}$$

The first term is the dipole-field interaction, the second one the self-energy of the dipoles and the last one the dipole-dipole interaction energy between different polarized atoms.

Combining Eqns. 2 and 3 and using supervector notation gives an equation for the induced polarization,

$$\boldsymbol{p} = \boldsymbol{\alpha}\left(\boldsymbol{f}[\rho] - \boldsymbol{T}\boldsymbol{p}\right), \tag{SI-8}$$

which is equivalent to

$$\left[\boldsymbol{\alpha}^{-1} + \boldsymbol{T}\right]\boldsymbol{p} = \boldsymbol{f}[\rho]. \tag{SI-9}$$

This matrix equation has the solution

$$\boldsymbol{p} = \boldsymbol{B}^{-1}\boldsymbol{f}[\rho] \qquad \text{with} \qquad \boldsymbol{B} = \boldsymbol{\alpha}^{-1} + \boldsymbol{T} \tag{SI-10}$$

Since $\boldsymbol{T}$ is symmetric and $\boldsymbol{\alpha}$ is diagonal, $\boldsymbol{B}$ is also symmetric. Plugging the polarization induced by the charge distribution (Eqn. SI-10) into the expression SI-7 for the polarization energy, yields the additional term that has to be added to the QM Hamiltonian:

$$\begin{aligned} U &= -\boldsymbol{f}^T\boldsymbol{B}^{-1}\boldsymbol{f} + \frac{1}{2}\boldsymbol{f}^T\left[\boldsymbol{B}^{-1}\underbrace{\left(\boldsymbol{\alpha}^{-1} + \boldsymbol{T}\right)}_{\boldsymbol{B}}\boldsymbol{B}^{-1}\right]\boldsymbol{f} \\ &= -\frac{1}{2}\boldsymbol{f}^T\boldsymbol{B}^{-1}\boldsymbol{f}. \end{aligned} \tag{SI-11}$$

The matrix

$$\boldsymbol{A} = \boldsymbol{B}^{-1} = \left(\boldsymbol{\alpha}^{-1} + \boldsymbol{T}\right)^{-1} \tag{SI-12}$$

can be understood as an effective dipole polarizability for the whole system. In the case of a single polarizable atom, $\boldsymbol{A}$ reduces to the atomic polarizability and we recover the energy of a single atom in a field $\boldsymbol{f}$: $U_0(\boldsymbol{p}) + U_1 = \frac{1}{2}\boldsymbol{p}^T\alpha^{-1}\boldsymbol{p} - \boldsymbol{f}^T\boldsymbol{p}$, where the first term is identified as the work required to polarize the atom and the second term is the electrostatic interaction of the resulting induced dipole and the field.

1.2 Damping functions

Dipole-dipole interaction. As pointed out by Thole,[2] the molecular polarizability tensor can have negative eigenvalues, so that the polarization energy diverges for certain geometric arrangements. This is an artifact of treating the dipole-dipole interaction classically. To avoid the polarization catastrophe[2] the dipole-dipole interaction has to be damped if two dipoles with polarizabilities α_1 and α_2 come close. For distances shorter than $s = 1.662(\alpha_1\alpha_2)^{1/6}$, the dipole-field tensor in Eqn. SI-1 is modified as[2]

$$\tilde{\boldsymbol{T}}(\boldsymbol{r}) = \begin{cases} 4v^3(1-v)r^{-3}\ \mathbb{1} + v^4\ \boldsymbol{T}(\boldsymbol{r}) & \text{if } r < s \\ \boldsymbol{T}(\boldsymbol{r}) & \text{if } r \geq s, \end{cases} \tag{SI-13}$$

where $v = r/s$.

Monopole-dipole interaction. The interaction between point charges and point dipoles is also problematic. The electric field generated by the nuclei and other point charges at the position of a polarizable atom diverges as the distance to any of the monopoles goes to zero. The field in Eqn. 16 of the main text becomes infinite if a point charge Q_n coincides with a polarizable atom at $\boldsymbol{R}$. The solution is to replace the dipole by a smeared out charge distribution.[2] The nuclear fields are multiplied by a damping function at short range (in addition to $C(r)$):

$$\tilde{\boldsymbol{F}}(R_i) = \sum_n Q_n \frac{\boldsymbol{R}_i - \boldsymbol{R}_n}{|\boldsymbol{R}_i - \boldsymbol{R}_n|^3}\ \lambda(|\boldsymbol{R}_i - \boldsymbol{R}_n|)C(|\boldsymbol{R}_i - \boldsymbol{R}_n|) \tag{SI-14}$$

with

$$\lambda(r) = \begin{cases} 4v^3 - 3v^4 & \text{if } r < s \\ 1 & \text{if } r \geq s \end{cases} \tag{SI-15}$$

The damping function $\lambda(r)$ ensures that the field vanishes as the two atoms fuse.

2 Detailed derivation of analytical gradients of integral exact direct reaction field

In the following, the gradient of the restricted Hartree-Fock (RHF) energy in combination with a QM/MM-IEDRF embedding scheme will be derived. According to Eqn. 4.25 in Ref. 3, the gradient of the plain RHF energy is given by

$$\frac{\partial E_{\mathrm{RHF}}}{\partial x} = \sum_{\mu,\nu}^{N_{\mathrm{AO}}} D_{\mu\nu} \frac{\partial H^{\mathrm{core}}_{\mu\nu}}{\partial x} - \sum_{\mu\nu}^{N_{\mathrm{AO}}} W_{\mu\nu} \frac{\partial S_{\mu\nu}}{\partial x} + \frac{1}{4} \sum_{\mu,\nu,\lambda,\sigma} \left\{ 2 D_{\mu\nu} D_{\lambda\sigma} - D_{\mu\lambda} D_{\nu\sigma} \right\} \frac{\partial (\mu\nu|\lambda\sigma)}{\partial x} + \frac{\partial V_{\mathrm{nuc}}}{\partial x}, \tag{SI-16}$$

where x represents any external parameter, which in our case could be the coordinates of the nuclei, the point charges or the polarizable sites. The gradient depends on the following quantities:

$$D_{\mu\nu} = \sum_{k \in \mathrm{occ}} 2 C^*_{\mu,k} C_{\nu,k} \tag{SI-17}$$

is the density matrix and

$$W_{\mu\nu} = \sum_{k \in \mathrm{occ}} 2 C^*_{\mu,k} C_{\nu,k} \varepsilon_k \tag{SI-18}$$

is the "energy-weighted" density matrix, both in the atomic orbitals basis. Here $C_{\mu,i}$ are the coefficients of the occupied molecular orbital i with orbital energy ε_i. Gradient terms of the form $\sum_{\mu,i} \partial E \big/ \partial C_{\mu,i} \; \partial C_{\mu,i} \big/ \partial x$ vanish, since the energy is variational with respect to the MO coefficients, $\partial E \big/ \partial C_{\mu,i} = 0$. $(\mu\nu|\lambda\sigma)$ are the two-electron repulsion integrals, $H^{\mathrm{core}}_{\mu\nu}$ and $S_{\mu\nu}$ are the core Hamiltonian and the overlap matrix, respectively, and V_{nuc} contains the nuclear energy. In order to limit the memory footprint of the algorithm, it is convenient to avoid storing large arrays with gradient of integrals. Instead gradients of matrix elements are immediately contracted with a density matrix. These contracted gradients are thus functions of up to two density matrices, labelled $\boldsymbol{D}^{(1)}$ and $\boldsymbol{D}^{(2)}$ for complete generality. After defining

the gradient contractions of the one-electron integrals,

$$\frac{\partial S}{\partial x}(\boldsymbol{D}) = \sum_{\mu\nu} \frac{\partial S_{\mu\nu}}{\partial x} D_{\mu\nu}, \tag{SI-19}$$

$$\frac{\partial H^{\text{core}}}{\partial x}(\boldsymbol{D}) = \sum_{\mu\nu} \frac{\partial H^{\text{core}}_{\mu\nu}}{\partial x} D_{\mu\nu}, \tag{SI-20}$$

and the Coulomb and exchange-like contractions

$$\frac{\partial J}{\partial x}(\boldsymbol{D}^{(1)}, \boldsymbol{D}^{(2)}) = \sum_{\mu,\nu,\lambda,\sigma} D^{(1)}_{\mu\nu} \frac{\partial(\mu\nu|\lambda\sigma)}{\partial x} D^{(2)}_{\lambda\sigma}, \tag{SI-21}$$

$$\frac{\partial K}{\partial x}(\boldsymbol{D}^{(1)}, \boldsymbol{D}^{(2)}) = \sum_{\mu,\nu,\lambda,\sigma} D^{(1)}_{\mu\lambda} \frac{\partial(\mu\nu|\lambda\sigma)}{\partial x} D^{(2)}_{\nu\sigma}, \tag{SI-22}$$

the gradient of the RHF energy can be expressed as

$$\frac{\partial E_{\text{RHF}}}{\partial x} = \frac{\partial H^{\text{core}}}{\partial x}(\boldsymbol{D}) - \frac{\partial S}{\partial x}(\boldsymbol{W}) + \frac{1}{2}\frac{\partial J}{\partial x}(\boldsymbol{D}, \boldsymbol{D}) - \frac{1}{4}\frac{\partial K}{\partial x}(\boldsymbol{D}, \boldsymbol{D}) + \frac{\partial V_{\text{nuc}}}{\partial x}. \tag{SI-23}$$

Although the focus will be on the RHF gradient, the functions in Eqns. SI-19 to SI-22 are in fact sufficient to express the energy gradient of many other wavefunction-based methods such as CIS or CASSCF, all be it using different generalized density matrices.

The polarization Hamiltonian does not depend on any reference state, it simply modifies the matrix elements of the molecular Hamiltonian. Therefore the gradient of the total RHF QM/MM-IEDRF energy follows directly from adding the gradients of the polarization Hamiltonian to the respective zero-, one- and two-electron terms. In the presence of a polarizable environment, the gradient of the J-like contraction, for instance, would have to be replaced by $\partial J \big/ \partial x + \partial(\Delta J^{\text{pol}}) \big/ \partial x$. The superscript ^{pol} will be dropped for brevity.

2.0.1 Gradients of Coulomb and Exchange Energy

Let us start with the gradient terms $\partial(\Delta J) \big/ \partial x$ and $\partial(\Delta K) \big/ \partial x$, as an efficient implementation of those is the most critical part. The polarization Hamiltonian reduces the

electron-electron repulsion integrals by the amount (see Eqn. 24)

$$(\mu\nu|\hat{h}^{(2)}(1,2)|\lambda\sigma) = -\boldsymbol{F}^{(e)}_{\mu\nu}\boldsymbol{A}\boldsymbol{F}^{(e)}_{\lambda\sigma}. \tag{SI-24}$$

Given two general (not necessarily symmetric) density matrices $\boldsymbol{D}^{(1)}$ and $\boldsymbol{D}^{(2)}$ the corrections to the Coulomb and exchange energies are

$$\Delta J(\boldsymbol{D}^{(1)},\boldsymbol{D}^{(2)}) = -\sum_{\mu,\nu,\lambda,\sigma} D^{(1)}_{\mu\nu}\left(\boldsymbol{F}^{(e)}_{\mu\nu}\boldsymbol{A}\boldsymbol{F}^{(e)}_{\lambda\sigma}\right)D^{(2)}_{\lambda\sigma}, \tag{SI-25}$$

$$\Delta K(\boldsymbol{D}^{(1)},\boldsymbol{D}^{(2)}) = -\sum_{\mu,\nu,\lambda,\sigma} D^{(1)}_{\mu\lambda}\left(\boldsymbol{F}^{(e)}_{\mu\nu}\boldsymbol{A}\boldsymbol{F}^{(e)}_{\lambda\sigma}\right)D^{(2)}_{\nu\sigma}. \tag{SI-26}$$

When taking derivatives of the RHF energy, the density matrices in the above expressions are kept constant, since their derivatives with respect to orbital coefficients do not need to be considered, and their derivatives with respect to the basis centers are already included as Pulay force terms. As a result, only gradients with respect to the matrix elements in brackets are needed. The gradient of those matrix elements follows from the product rule of differentiation:

$$\frac{\partial}{\partial x}(\mu\nu|\hat{h}^{(2)}(1,2)|\lambda\sigma) = -\frac{\partial \boldsymbol{F}^{(e)}_{\mu\nu}}{\partial x}\boldsymbol{A}\boldsymbol{F}^{(e)}_{\lambda\sigma} - \boldsymbol{F}^{(e)}_{\mu\nu}\frac{\partial \boldsymbol{A}}{\partial x}\boldsymbol{F}^{(e)}_{\lambda\sigma} - \boldsymbol{F}^{(e)}_{\mu\nu}\boldsymbol{A}\frac{\partial \boldsymbol{F}^{(e)}_{\lambda\sigma}}{\partial x} \tag{SI-27}$$

To find the gradient of the effective polarizability tensor $\boldsymbol{A}$ one notes that

$$0 = \frac{\partial}{\partial x}\mathbb{1} = \frac{\partial}{\partial x}\left(\boldsymbol{A}\boldsymbol{A}^{-1}\right) = \frac{\partial \boldsymbol{A}}{\partial x}\boldsymbol{A}^{-1} + \boldsymbol{A}\frac{\partial \boldsymbol{A}^{-1}}{\partial x}, \tag{SI-28}$$

which can be solved for

$$\frac{\partial \boldsymbol{A}}{\partial x} = -\boldsymbol{A}\frac{\partial \boldsymbol{A}^{-1}}{\partial x}\boldsymbol{A}. \tag{SI-29}$$

Since $\boldsymbol{A}^{-1} = \boldsymbol{\alpha}^{-1} + \boldsymbol{T}$ and the atomic polarizabilities $\boldsymbol{\alpha}$ are constant, we get

$$\frac{\partial \boldsymbol{A}}{\partial x} = -\boldsymbol{A}\frac{\partial \boldsymbol{T}}{\partial x}\boldsymbol{A}. \tag{SI-30}$$

The full gradient of $\boldsymbol{A}$ is not kept in memory, derivatives with respect to individual coordinates are computed as needed. Since the effective polarizability tensor only depends on the polarizable site, the gradient $\partial \boldsymbol{A} \big/ \partial x$ is zero, if x is a QM atom or a point charge.

When computing the additional J- and K-like contractions due to the polarization Hamiltonian, the order of the summation is important. Round brackets are used to show the presumably best order, which allows to contract gradients of matrix elements with the corresponding elements in the density matrix on the fly. Written out with all indices, the polarization Hamiltonian's contribution to the J-like contraction takes the form

$$\begin{aligned}\frac{\partial \Delta J}{\partial x}(\boldsymbol{D}^{(1)}, \boldsymbol{D}^{(2)}) = &-\sum_i \sum_{\mu,\nu} \frac{\partial F^{(e)}_{i,\mu\nu}}{\partial x}\left(\sum_j A_{ij}\left(\sum_{\lambda,\sigma} F^{(e)}_{j,\lambda\sigma} D^{(2)}_{\lambda\sigma}\right)\right) D^{(1)}_{\mu\nu} \\ &-\sum_{i,j}\left(\sum_{\mu,\nu} F^{(e)}_{i,\mu\nu} D^{(1)}_{\mu\nu}\right)\frac{\partial A_{ij}}{\partial x}\left(\sum_{\lambda,\sigma} F_{j,\lambda\sigma} D^{(2)}_{\lambda\sigma}\right) \\ &-\sum_i \sum_{\mu,\nu} \frac{\partial F^{(e)}_{i,\mu\nu}}{\partial x}\left(\sum_j A_{ij}\left(\sum_{\lambda\sigma} F^{(e)}_{j,\lambda\sigma} D^{(1)}_{\lambda\sigma}\right)\right) D^{(2)}_{\mu\nu}.\end{aligned} \tag{SI-31}$$

The placing of brackets suggests that the following vectors should be stored as intermediates during the evaluation of the J-like gradient contractions:

$$(FD)_i = \sum_{\mu\nu} F^{(e)}_{i,\mu\nu} D_{\mu\nu} \tag{SI-32}$$

$$(AFD)_i = \sum_j A_{ij} (FD)_j \tag{SI-33}$$

where $\boldsymbol{D}$ stands for any density matrix. In addition, the contraction of the derivative polarization integrals, $\partial \boldsymbol{F}^{(e)} \big/ \partial x$, with a tensor $\boldsymbol{E}$ of dimensions $(3N_{\mathrm{pol}}) \times N_{\mathrm{AO}} \times N_{\mathrm{AO}}$ has to be

computed in an integral-direct fashion:

$$\frac{\partial F}{\partial x}(\boldsymbol{E}) = \sum_i \sum_{\mu,\nu} \frac{\partial F^{(e)}_{i,\mu\nu}}{\partial x} E_{i,\mu\nu}. \tag{SI-34}$$

The derivative polarization integrals are evaluated inside the loop over i, μ and ν. They are directly multiplied with the corresponding matrix element of the density matrix and are added to the gradient $\partial F \big/ \partial x$. Similarly we define the contraction of a $(3N_{\text{pol}}) \times (3N_{\text{pol}})$ matrix $\boldsymbol{U}$ with the gradient of the effective polarizability,

$$\frac{\partial A}{\partial x}(\boldsymbol{U}) = \sum_{i,j} \frac{\partial A_{ij}}{\partial x} U_{ij} \tag{SI-35}$$

In terms of the intermediate vectors and the functions $\partial F \big/ \partial x$ and $\partial A \big/ \partial x$ the additional gradient due to the Coulomb interaction of two density matrices via the polarizable medium becomes

$$\begin{aligned} \frac{\partial(\Delta J)}{\partial x}(\boldsymbol{D}^{(1)}, \boldsymbol{D}^{(2)}) = &- \frac{\partial F}{\partial x}\Big((\boldsymbol{AFD}^{(2)}) \otimes \boldsymbol{D}^{(1)} + (\boldsymbol{AFD}^{(1)}) \otimes \boldsymbol{D}^{(2)} \Big) \\ &- \frac{\partial A}{\partial x}\left((\boldsymbol{FD}^{(1)}) \otimes (\boldsymbol{FD}^{(2)}) \right). \end{aligned} \tag{SI-36}$$

Here, the argument of the function $\partial F \big/ \partial x$ contains Kronecker products between a vector of size N_{pol} and a density matrix of dimensions $N_{\text{AO}} \times N_{\text{AO}}$, e.g.

$$\left[(\boldsymbol{AFD}^{(2)} \otimes \boldsymbol{D}^{(1)} \right]_{i,\mu\nu} = \left(AFD^{(2)} \right)_i D^{(1)}_{\mu\nu}. \tag{SI-37}$$

The contraction of the gradient of the exchange operator with another density matrix,

$$\frac{\partial(\Delta K)}{\partial x}(\boldsymbol{D}^{(1)}, \boldsymbol{D}^{(2)}) = \sum_{\mu,\nu,\lambda,\sigma} D^{(1)}_{\mu\lambda} \frac{\partial(\mu\nu|h^{(2)}|\lambda\sigma)}{\partial x} D^{(2)}_{\nu\sigma}, \tag{SI-38}$$

is probably best computed in the following order:

$$\begin{aligned}
\frac{\partial(\Delta K)}{\partial x} = &-\sum_i \sum_{\mu,\nu} \frac{F^{(e)}_{1,\mu\nu}}{\partial x} \left(\sum_j A_{ij} \left(\sum_\lambda \left(\sum_\sigma F^{(e)}_{j,\lambda\sigma} (D^{(2)T})_{\sigma\nu} \right) (D^{(1)T})_{\lambda\mu} \right) \right) \\
&- \sum_{i,j} \frac{\partial A_{ij}}{\partial x} \left(\sum_{\mu,\nu} F^{(e)}_{i,\mu\nu} \left(\sum_\lambda \left(\sum_\sigma F^{(e)}_{j,\lambda\sigma} (D^{(2)T})_{\sigma\nu} \right) (D^{(1)T})_{\lambda\mu} \right) \right) \\
&- \sum_i \sum_{\mu,\nu} \frac{\partial F^{(e)}_{i,\mu\nu}}{\partial x} \left(\sum_j A_{ij} \left(\sum_\lambda \left(\sum_\sigma F^{(e)}_{j,\lambda\sigma} D^{(2)}_{\sigma\nu} \right) D^{(1)}_{\lambda\mu} \right) \right)
\end{aligned} \tag{SI-39}$$

$(D^T)_{\mu\nu} = D_{\nu\mu}$ are the matrix elements of the transpose of $\boldsymbol{D}$ and the symmetry $A_{ij} = A_{ji}$ was used to bring the first and third line into a similar form (except for the transposition of the density matrices). The order of computation has been chosen such that the intermediates that exist in memory at any time fit into one or two arrays of size $(3N_{\text{pol}}) \times N_{\text{AO}} \times N_{\text{AO}}$. The intermediates arise from transforming each of the three indices of $F^{(e)}_{j,\mu\nu}$ to another basis. Transforming the third and the second indices by the density matrices and the first index by the $\boldsymbol{A}$ matrix is done in three steps:

$$(FD^{(2)})_{j,\lambda\nu} = \sum_\sigma F^{(e)}_{j,\lambda\sigma} D^{(2)}_{\sigma\nu} \tag{SI-40}$$

$$(FD^{(2)}D^{(1)})_{j,\mu\nu} = \sum_\lambda (FD^{(2)})_{j,\lambda\nu} D^{(1)}_{\lambda\mu} \tag{SI-41}$$

$$(AFD^{(2)}D^{(1)})_{i,\mu\nu} = \sum_j A_{ij} (FD^{(2)}D^{(1)})_{j,\mu\nu}, \tag{SI-42}$$

and similary for the transposes of the density matrices,

$$(FD^{(2)T})_{j,\lambda\nu} = \sum_\sigma F^{(e)}_{j,\lambda\sigma} D^{(2)}_{\nu\sigma} \tag{SI-43}$$

$$(FD^{(2)T}D^{(1)T})_{j,\mu\nu} = \sum_\lambda (FD^{(2)T})_{j,\lambda\nu} D^{(1)}_{\mu\lambda} \tag{SI-44}$$

$$(AFD^{(2)T}D^{(1)T})_{i,\mu\nu} = \sum_j A_{ij} (FD^{(2)T}D^{(1)T})_{j,\mu\nu}. \tag{SI-45}$$

Contracting Eqn. SI-44 with the polarization integrals gives

$$(FFD^{(2)T}D^{(1)T})_{ij} = \sum_{\mu\nu} F^{(e)}_{i,\mu\nu}(FD^{(2)T}D^{(1)T})_{j,\mu\nu}. \tag{SI-46}$$

Using the just defined quantities, the exchange part of the gradient becomes

$$\frac{\partial(\Delta K)}{\partial x}(\boldsymbol{D}^{(1)}, \boldsymbol{D}^{(2)}) = -\frac{\partial F}{\partial x}\Big(\boldsymbol{AFD^{(2)}D^{(1)}} + \boldsymbol{AFD^{(2)T}D^{(1)T}}\Big) - \frac{\partial A}{\partial x}\left(\boldsymbol{FFD^{(2)T}D^{(1)T}}\right). \tag{SI-47}$$

2.0.2 Gradient of Core Hamiltonian

Now let us turn our attention to the gradients of the core Hamiltonian,

$$\frac{\partial(\Delta H^{\text{core}})}{\partial x}(\boldsymbol{D}) = \sum_{\mu,\nu} \frac{\partial(\mu|\hat{h}^{(1)}|\nu)}{\partial x} D_{\mu\nu}. \tag{SI-48}$$

The matrix elements of $\hat{h}^{(1)}$ are given in Eqn. 26 . Each term is treated separately. The gradient belonging to the interaction between the nuclear and the electronic fields is computed as

$$\begin{aligned}\sum_{\mu,\nu} \frac{\partial}{\partial x}\left(-\boldsymbol{F}^{(n)}\boldsymbol{A}\boldsymbol{F}^{(e)}_{\mu\nu}\right) D_{\mu\nu} = &-\sum_{i,j} \frac{\partial F^{(n)}_i}{\partial x} A_{ij} \left(\sum_{\mu,\nu} F^{(e)}_{j,\mu\nu} D_{\mu\nu}\right) \\ &-\sum_{i,j} F^{(n)}_i \frac{\partial A_{ij}}{\partial x} \left(\sum_{\mu,\nu} F^{(e)}_{j,\mu\nu} D_{\mu\nu}\right) \\ &-\sum_{i}\sum_{\mu,\nu} \frac{\partial F^{(e)}_{i,\mu\nu}}{\partial x} \left(\left(\sum_{j} A_{ij} F^{(n)}_j\right) D_{\mu\nu}\right).\end{aligned} \tag{SI-49}$$

After defining the intermediate vectors

$$(FD)_i = \sum_{\mu,\nu} F^{(e)}_{i,\mu\nu} D_{\mu\nu} \tag{SI-50}$$

$$(AF^{(n)})_i = \sum_j A_{ij} F^{(n)}_j \tag{SI-51}$$

the first term becomes

$$\sum_{\mu,\nu} \frac{\partial}{\partial x} \left(-\boldsymbol{F}^{(n)} \boldsymbol{A} \boldsymbol{F}^{(e)}_{\mu\nu} \right) D_{\mu\nu} = -\sum_{i,j} \left(\frac{\partial F^{(n)}_i}{\partial x} A_{ij} + F^{(n)}_i \frac{\partial A_{ij}}{\partial x} \right) (FD)_j - \frac{\partial F}{\partial x} \left((\boldsymbol{A}\boldsymbol{F}^{(\boldsymbol{n})} \otimes \boldsymbol{D} \right) \tag{SI-52}$$

where again use is made of the function $\partial F \big/ \partial x \ (\boldsymbol{U})$ defined in Eqn. SI-34. The second term of Eqn. 26 may be expressed via the function $\Delta K(\cdot,\cdot)$ (see Eqn. SI-26) as

$$-\frac{1}{2} \sum_{\mu,\nu,\lambda,\sigma} D_{\mu\lambda} \boldsymbol{F}^{(e)}_{\mu\nu} \boldsymbol{A} \boldsymbol{F}^{(e)}_{\lambda\sigma} (\boldsymbol{S}^{-1})_{\nu\sigma} = \frac{1}{2} \Delta K \left(\boldsymbol{D}, \boldsymbol{S}^{-1} \right). \tag{SI-53}$$

Since the overlap matrix depends on the position of the QM atoms, the gradient of previous expression becomes

$$-\frac{1}{2} \sum_{\mu,\nu,\lambda,\sigma} \frac{\partial}{\partial x} \left(\boldsymbol{F}^{(e)}_{\mu\lambda} \boldsymbol{A} \boldsymbol{F}^{(e)}_{\nu\sigma} (\boldsymbol{S}^{-1})_{\lambda\sigma} \right) D_{\mu\nu} = \frac{1}{2} \frac{\partial(\Delta K)}{\partial x} (\boldsymbol{D}, \boldsymbol{S}^{-1}) + \frac{1}{2} \Delta K \left(\boldsymbol{D}, \frac{\partial \boldsymbol{S}^{-1}}{\partial x} \right), \tag{SI-54}$$

where the functions ΔK and $\partial(\Delta K) \big/ \partial x$ are defined in Eqns. SI-26 and SI-47, respectively. The gradient of the inverse overlap matrix is formally expressed as

$$\frac{\partial \boldsymbol{S}^{-1}}{\partial x} = -\boldsymbol{S}^{-1} \frac{\partial \boldsymbol{S}}{\partial x} \boldsymbol{S}^{-1} \tag{SI-55}$$

but it is not convenient to compute it in this way and store it entirely in memory. Instead $\Delta K(\boldsymbol{D}, \partial \boldsymbol{S}^{-1} \big/ \partial x)$ should be formulated as a contraction of the overlap gradients with some matrix, which can be evaluated with the help of the function $\partial S \big/ \partial x$ defined in Eqn. SI-19.

To this end the factors have to be reordered:

$$\frac{1}{2}\Delta K\left(\boldsymbol{D},\frac{\partial \boldsymbol{S}^{-1}}{\partial x}\right)=\frac{1}{2}\sum_{\alpha,\beta}\frac{\partial S_{\alpha\beta}}{\partial x}\sum_{i}\sum_{\lambda}\left(\sum_{\mu}\left(\sum_{\nu}F^{(e)}_{i,\mu\nu}(\boldsymbol{S}^{-1})_{\nu\alpha}\right)D_{\mu\lambda}\right)\times\left(\sum_{j}A_{ij}\left(\sum_{\sigma}F^{(e)}_{j,\lambda\sigma}(\boldsymbol{S}^{-1})_{\sigma\beta}\right)\right) \quad \text{(SI-56)}$$

The necessary intermediate tensors result from transforming two of the three indices of the polarization integrals $\boldsymbol{F}^{(e)}$:

$$(FS^{-1})_{i,\mu\alpha}=\sum_{\nu}F^{(e)}_{i,\mu\nu}(\boldsymbol{S}^{-1})_{\nu\alpha} \quad \text{(SI-57)}$$

$$(FS^{-1}D)_{i,\lambda\alpha}=\sum_{\mu}(FS^{-1})_{i,\mu\alpha}D_{\mu\lambda} \quad \text{(SI-58)}$$

$$(AFS^{-1})_{i,\lambda\beta}=\sum_{j}A_{ij}(FS^{-1})_{j,\lambda\beta} \quad \text{(SI-59)}$$

The memory requirements are at least two arrays of size $(3N_{\text{pol}})\times N_{\text{AO}}\times N_{\text{AO}}$. The tensors in Eqns. SI-58 and SI-59 are summed over the first two indices to give

$$Q_{\alpha\beta}=\sum_{i,\lambda}(FS^{-1}D)_{i,\lambda\alpha}(AFS^{-1})_{i,\lambda\beta}. \quad \text{(SI-60)}$$

Then Eqn. SI-54 turns into

$$\begin{aligned}-\frac{1}{2}\sum_{\mu,\nu,\lambda,\sigma}\frac{\partial}{\partial x}\left(\boldsymbol{F}^{(e)}_{\mu\lambda}\boldsymbol{A}\boldsymbol{F}^{(e)}_{\nu\sigma}(\boldsymbol{S}^{-1})_{\lambda\sigma}\right)D_{\mu\nu}=&\frac{1}{2}\frac{\partial(\Delta K)}{\partial x}\left(\boldsymbol{D},\boldsymbol{S}^{-1}\right)+\frac{1}{2}\frac{\partial S}{\partial x}(\boldsymbol{Q})\\=&-\frac{\partial F}{\partial x}\left(\frac{1}{2}\left[\boldsymbol{AFS^{-1}D}+\boldsymbol{AFS^{-1}D^{T}}\right]\right)+\frac{1}{2}\frac{\partial S}{\partial x}(\boldsymbol{Q})\\&-\frac{1}{2}\sum_{i,j}\frac{\partial A_{ij}}{\partial x}(FFS^{-1}D^{T})_{ij}\end{aligned} \quad \text{(SI-61)}$$

Of course, when derivatives are taken with respect to the polarizable sites or point charges, the gradient of the overlap matrix is zero and the second term can be dropped.

The third and fourth terms in Eqn. 26 are only needed if same site integrals are to be evaluated exactly. In this case the contracted gradient of the third term is evaluated analogously to the second one but replacing $\boldsymbol{A}$ with a the block diagonal matrix $\mathrm{diag}(\boldsymbol{A})_{3\times3}$,

$$\begin{aligned}\frac{1}{2}\sum_{\mu,\nu,\lambda,\sigma}\frac{\partial}{\partial x}\left(\boldsymbol{F}^{(e)}_{\mu\lambda}\,\mathrm{diag}(\boldsymbol{A})_{3\times3}\boldsymbol{F}^{(e)}_{\nu\sigma}(\boldsymbol{S}^{-1})_{\lambda\sigma}\right)D_{\mu\nu} = &-\frac{1}{2}\frac{\partial(\Delta K)}{\partial x}\Big|_{\boldsymbol{A}=\mathrm{diag}(\boldsymbol{A})_{3\times3}}(\boldsymbol{D},\boldsymbol{S}^{-1})\\ &-\frac{1}{2}\frac{\partial S}{\partial x}\left(\boldsymbol{Q}\Big|_{\boldsymbol{A}=\mathrm{diag}(\boldsymbol{A})_{3\times3}}\right)\end{aligned} \tag{SI-62}$$

Note that the gradients of the diagonal blocks of $\boldsymbol{A}$ are in general nonzero.

Finally the fourth term contains the gradients of the exact same-site integrals of Eqn. 20 , $I^{\alpha\beta}_{k,\mu\nu} = I^{\alpha\beta}_{\mu\mu}(\boldsymbol{R}_k)$ with $k = 1,\ldots,N_{\mathrm{pol}}$ and $\alpha,\beta = 1,2,3$. The gradient contraction of the fourth term is

$$\begin{aligned}-\frac{1}{2}\sum_{\mu\nu}\sum_{k=1}^{N_{\mathrm{pol}}}\sum_{\alpha,\beta=1}^{3}\frac{\partial}{\partial x}\left(A^{\alpha\beta}_{kk}I^{\alpha\beta}_{k,\mu\nu}\right)D_{\mu\nu} = -\frac{1}{2}\sum_{k=1}^{N_{\mathrm{pol}}}\sum_{\alpha,\beta=1}^{3}\Bigg(&\frac{\partial A^{\alpha\beta}_{kk}}{\partial x}\sum_{\mu\nu}I^{\alpha\beta}_{k,\mu\nu}D_{\mu\nu}\\ &+A^{\alpha\beta}_{kk}\sum_{\mu\nu}\frac{\partial I^{\alpha\beta}_{k,\mu\nu}}{\partial x}D_{\mu\nu}\Bigg).\end{aligned} \tag{SI-63}$$

We define an intermediate tensor of dimension $(N_{\mathrm{pol}}\times3\times3)$,

$$(ID)_{k,\alpha\beta} = \sum_{\mu,\nu}I^{\alpha\beta}_{k,\mu\nu}D_{\mu\nu}, \tag{SI-64}$$

$$\tag{SI-65}$$

and another tensor of dimensions $(N_{\mathrm{pol}}\times3\times3\times N_{\mathrm{AO}}\times N_{\mathrm{AO}})$,

$$Y_{k,\alpha\beta,\mu\nu} = A^{\alpha\beta}_{kk}D_{\mu\nu}, \tag{SI-66}$$

as well as a function for evaluating contractions of the gradients of the I-integrals,

$$\frac{\partial I}{\partial x}(\boldsymbol{Y}) = \sum_{k=1}^{N_{\mathrm{pol}}} \sum_{\alpha,\beta=1}^{3} \sum_{\mu,\nu=1}^{N_{\mathrm{AO}}} \frac{\partial I_{k,\mu\nu}^{\alpha\beta}}{\partial x} Y_{k,\alpha\beta,\mu\nu} \tag{SI-67}$$

With these definitions Eqn. SI-63 becomes

$$-\frac{1}{2}\sum_{\mu\nu}\sum_{k=1}^{N_{\mathrm{pol}}}\sum_{\alpha,\beta=1}^{3} \frac{\partial}{\partial x}\left(A_{kk}^{\alpha\beta} I_{k,\mu\nu}^{\alpha\beta}\right) D_{\mu\nu} = -\frac{1}{2}\sum_{k=1}^{N_{\mathrm{pol}}}\sum_{\alpha,\beta=1}^{3} \frac{\partial A_{kk}^{\alpha\beta}}{\partial x}(ID)_{k,\alpha\beta} - \frac{1}{2}\frac{\partial I}{\partial x}(\boldsymbol{Y}). \tag{SI-68}$$

Since the function $\partial F \big/ \partial x$ is linear, Eqns. SI-52 and SI-61 can be combined. In order to incorporate also Eqns. SI-62 and SI-68, we define the flag ξ, which is 0 if all integrals are evaluated with the resolution of identity or 1 if same-site integrals are evaluated exactly. The polarizability tensor is then replaced by

$$\bar{\boldsymbol{A}} = \boldsymbol{A} - \xi \,\mathrm{diag}(\boldsymbol{A})_{3\times 3} \quad \text{with} \quad \xi \in \{0,1\} \tag{SI-69}$$

Now all the pieces are put together. To group the terms into meaningful entities, the gradient of the core Hamiltonian is decomposed into the sum of the gradients of its constituents by using the chain rule:

$$\frac{\partial \Delta \boldsymbol{H}^{\mathrm{core}}}{\partial x}\bigg|_{\boldsymbol{D}} = \frac{\partial \boldsymbol{A}}{\partial x}\cdot\frac{\partial \boldsymbol{H}}{\partial \boldsymbol{A}} + \xi\frac{\partial\,\mathrm{diag}(\boldsymbol{A})}{\partial x}\cdot\frac{\partial \boldsymbol{H}}{\partial\,\mathrm{diag}(\boldsymbol{A})} + \frac{\partial \boldsymbol{F}^{(n)}}{\partial x}\cdot\frac{\partial \boldsymbol{H}}{\partial \boldsymbol{F}^{(n)}} + \frac{\partial \boldsymbol{F}^{(e)}}{\partial x}\cdot\frac{\partial \boldsymbol{H}}{\partial \boldsymbol{F}^{(e)}} + \xi\frac{\partial \boldsymbol{I}}{\partial x}\cdot\frac{\partial \boldsymbol{H}}{\partial \boldsymbol{I}} + \frac{\partial \boldsymbol{S}}{\partial x}\cdot\frac{\partial \boldsymbol{H}}{\partial \boldsymbol{S}} \tag{SI-70}$$

The density matrix is held constant. Each term is understood as a contraction of a gradient with respect to x and a tensor. The contraction has to be implemented as a linear function, where gradients of integrals are computed on the fly and are directly combined with the corresponding elements of the tensor. Below the definitions of those linear functions and tensors will be presented for each term. But first the scalar products in Eqn. SI-70 are replaced by linear functions (these have to be implemented as GPU kernels), which take the

partial derivatives of the core Hamiltonian as inputs:

$$\frac{\partial \Delta \boldsymbol{H}^{\text{core}}}{\partial x}\Big|_{\boldsymbol{D}} = \frac{\partial A}{\partial x}(\boldsymbol{U})+\xi\frac{\partial\,\text{diag}(A)}{\partial x}(\boldsymbol{V})+\frac{\partial F^{(n)}}{\partial x}(\boldsymbol{N})+\frac{\partial F^{(e)}}{\partial x}(\boldsymbol{E})+\xi\frac{\partial I}{\partial x}(\boldsymbol{Y})+\frac{\partial S}{\partial x}(\boldsymbol{R}) \quad \text{(SI-71)}$$

All the tensors are functions of the density matrix. The meanings and ranges of the tensor indices are summarized below:

- atomic orbials: $\mu, \nu, \lambda, \sigma, \gamma, \delta = 1, \ldots, N_{\text{AO}}$
- coordinates of polarizable sites: $i, j = 1, \ldots, 3N_{\text{pol}}$
- polarizable sites: $k = 1, \ldots, N_{\text{pol}}$
- Cartesian axes: $\alpha, \beta = 1, 2, 3$

1. The gradient of the effective polarizability tensor,

$$\frac{\partial A}{\partial x}(\boldsymbol{U}) = \sum_{i,j} \frac{\partial A_{ij}}{\partial x} U_{ij}, \quad \text{(SI-72)}$$

takes a $(3N_{\text{pol}}) \times (3N_{\text{pol}})$ tensor as input,

$$U_{ij}(\boldsymbol{D}) = -F_i^{(n)} \sum_{\mu,\nu} F^{(e)}_{j,\mu\nu} D_{\mu\nu} - \frac{1}{2}\sum_{\mu,\nu} F^{(e)}_{i,\mu\nu}\left(\sum_{\lambda}\left(\sum_{\sigma} F^{(e)}_{j,\lambda\sigma}(S^{-1})_{\sigma\nu}\right) D_{\mu\lambda}\right). \quad \text{(SI-73)}$$

2. The gradient of the diagonal blocks of the polarizability tensor,

$$\frac{\partial\,\text{diag}(A)}{\partial x}(\boldsymbol{V}) = \sum_{k}\sum_{\alpha,\beta} \frac{\text{diag}(A)^{\alpha\beta}_{kk}}{\partial x} V_{k,\alpha\beta} \quad \text{(SI-74)}$$

takes a $N_{\text{pol}} \times 3 \times 3$ tensor as input,

$$V_{k,\alpha\beta}(\boldsymbol{D}) = \frac{1}{2}\sum_{\mu,\nu} F^{(e)}_{3k+\alpha,\mu\nu}\left(\sum_{\lambda}\left(\sum_{\sigma} F^{(e)}_{3k+\beta,\lambda\sigma}(S^{-1})_{\sigma\nu}\right) D_{\mu\lambda}\right) - \frac{1}{2}\sum_{\mu,\nu} I^{\alpha\beta}_{k,\mu\nu} D_{\mu\nu} \quad \text{(SI-75)}$$

3. The gradient due to the nuclear fields,

$$\frac{\partial F^{(n)}}{\partial x}(\boldsymbol{N}) = \sum_i \frac{\partial F_i^{(n)}}{\partial x} N_i, \tag{SI-76}$$

takes a $(3N_{\mathrm{pol}})$ sized vector as input,

$$N_i(\boldsymbol{D}) = -\sum_j A_{ij} \sum_{\mu,\nu} F^{(e)}_{j,\mu\nu} D_{\mu\nu}. \tag{SI-77}$$

4. The gradient due to the electronic fields,

$$\frac{\partial F^{(e)}}{\partial x}(\boldsymbol{E}) = \sum_i \sum_{\mu,\nu} \frac{\partial F^{(e)}_{i,\mu\nu}}{\partial x} E_{i,\mu\nu} \tag{SI-78}$$

takes a $(3N_{\mathrm{pol}}) \times N_{\mathrm{AO}} \times N_{\mathrm{AO}}$ tensor as input,

$$E_{i,\mu\nu}(\boldsymbol{D}) = -\left(\sum_j A_{ij} F_j^{(n)}\right) D_{\mu\nu} - \frac{1}{2}\sum_j \bar{A}_{ij} \left(\sum_\lambda \left(\sum_\sigma F^{(e)}_{j,\lambda\sigma} (S^{-1})_{\sigma\nu}\right) [D_{\mu\lambda} + D_{\lambda\mu}]\right). \tag{SI-79}$$

5. The gradient due to the exact same-site integrals,

$$\frac{\partial I}{\partial x}(\boldsymbol{Y}) = \sum_k \sum_{\alpha,\beta} \sum_{\mu,\nu} \frac{\partial I^{\alpha\beta}_{k,\mu\nu}}{\partial x} Y_{k,\alpha\beta,\mu\nu} \tag{SI-80}$$

takes a $N_{\mathrm{pol}} \times 3 \times 3 \times N_{\mathrm{AO}} \times N_{\mathrm{AO}}$ sized vector as input,

$$Y_{k,\alpha\beta,\mu\nu}(\boldsymbol{D}) = -\frac{1}{2} A^{\alpha\beta}_{kk} D_{\mu\nu} \tag{SI-81}$$

6. The gradient due to the coordinate dependence of the overlap matrix,

$$\frac{\partial S}{\partial x}(\boldsymbol{R}) = \sum_{\mu,\nu} \frac{\partial S_{\mu\nu}}{\partial x} R_{\mu\nu}, \tag{SI-82}$$

takes a $N_{\text{AO}} \times N_{\text{AO}}$ sized matrix as input,

$$R_{\mu\nu}(\boldsymbol{D}) = \frac{1}{2}\sum_i \sum_\lambda \left[\sum_\gamma \left(\sum_\delta F^{(e)}_{i,\gamma\delta}(S^{-1})_{\delta\mu}\right) D_{\gamma\lambda}\right] \left[\sum_j \bar{A}_{ij} \sum_\delta F^{(e)}_{j,\lambda\delta}(S^{-1})_{\delta\nu}\right] \quad \text{(SI-83)}$$

2.0.3 Nuclear Gradients

The gradient of the 0-electron part of the polarization Hamiltonian,

$$h^{(0)} = -\frac{1}{2}\sum_{i,j} F^{(n)}_i A_{ij} F^{(n)}_j, \quad \text{(SI-84)}$$

takes the form

$$\frac{\partial h^{(0)}}{\partial x} = \frac{\partial F^{(n)}}{\partial x}(\boldsymbol{K}) + \frac{\partial A}{\partial x}(\boldsymbol{L}) \quad \text{(SI-85)}$$

with

$$K_i = -\sum_j A_{ij} F^{(n)}_j \quad \text{(SI-86)}$$

$$L_{ij} = -\frac{1}{2} F^{(n)}_i F^{(n)}_j. \quad \text{(SI-87)}$$

In all the above expressions the term $\frac{\partial A}{\partial x}(\boldsymbol{U})$ can be further simplified by noting that

$$\begin{aligned}
\frac{\partial A}{\partial x}(\boldsymbol{U}) &= \sum_{i,j} \frac{\partial A_{ij}}{\partial x} U_{ij} = -\sum_{i,j}\left(\boldsymbol{A}\frac{\partial T}{\partial x}\boldsymbol{A}\right)_{ij} U_{ij} \\
&= -\sum_{i,j,k,l} A_{ik}\frac{\partial T_{kl}}{\partial x} A_{lj} U_{ij} \\
&= -\sum_{k,l}\frac{\partial T_{kl}}{\partial x}\left(\sum_{i,j} A_{ki} U_{ij} A_{jl}\right) \\
&= -\frac{\partial T}{\partial x}(\boldsymbol{AUA})
\end{aligned} \quad \text{(SI-88)}$$

where $i, j, k, l = 1, \ldots, 3N_{\text{pol}}$. We have defined a new contraction of the gradient of the dipole field tensor $\partial \boldsymbol{T} \big/ \partial x$ with a $(3N_{\text{pol}}) \times (3N_{\text{pol}})$ matrix $\boldsymbol{AUA}$.

The correctness of the implementation was verified by comparing the analytical gradients of the total (excited state) energy with a 5th order finite-difference approximation. The non-adiabatic coupling vectors were checked similarly.

3 Protocol for Optimal Tuning of the Range-Separation Parameter

For the TD-DFT excited state calculations, the optimally-tuned version of Rohrdanz' ωPBEh functional[4] with $C_{\mathrm{HF}} = 0.2$ is employed:

$$E_{\mathrm{xc}}^{\omega\mathrm{PBEh}}(\omega) = E_{c,\mathrm{PBE}} + (1 - C_{\mathrm{HF}})E_{x,\mathrm{PBE}}^{\mathrm{SR}}(\omega) + C_{\mathrm{HF}}E_{x,\mathrm{HF}}^{\mathrm{FR}} + (1 - C_{\mathrm{HF}})E_{x,\mathrm{HF}}^{\mathrm{LR}}(\omega) \quad \text{(SI-89)}$$

ω is a fitting parameter that determines where the Coulomb operator is split into the short-range (SR) and the long-range (LR) parts. In $E_{x,\mathrm{HF}}^{\mathrm{FR}}$, the Hartree-Fock exchange is applied to the full-range (FR) potential.

Following Ref. 5, the optimal value of ω was determined by performing ground state DFT calculations (ωPBEh/aug-cc-pVDZ) for each molecule with $N-1$ (cation), N (neutral) and $N+1$ (anion) electrons for a range of ω values. For the exact functional of the density, minus the HOMO energy should equal the ionization potential (IP) (Janak's theorem).[6] Enforcing this condition both for the neutral and the anion, gives the following objective function:[5]

$$\begin{aligned} J^2(\omega) &= |E_{\mathrm{HOMO}}(N,\omega) + \mathrm{IP}(N,\omega)|^2 + |E_{\mathrm{HOMO}}(N+1,\omega) + \mathrm{IP}(N+1,\omega)|^2 \\ &= |E_{\mathrm{HOMO}}(N,\omega) + E(N-1,\omega) - E(N,\omega)|^2 + |E_{\mathrm{HOMO}}(N+1,\omega) + E(N,\omega) - E(N+1,\omega)|^2 \end{aligned} \quad \text{(SI-90)}$$

The range-separation parameters were tuned separately in vacuum and in solution. In the latter case the embedding Hamiltonian (either QM/MM or QM/MM+IEDRF) was included. For the dye 7c we repeated the tuning for all optimized snapshots, but only found a very

weak dependence on the solvent configurations (less than 0.005 Bohr^{-1}). Therefore, the optimal range-separation parameter was determined from a single snapshot for each dye.

A coarse grid covering the range $0.0 \leq \omega \leq 1.0$ Bohr^{-1} in equidistant steps of size $\Delta\omega = 0.05$ Bohr^{-1} was used to determine the approximate minimum of $J^2(\omega)$, before switching to a finer grid with resolution $\Delta\omega = 0.01$ Bohr^{-1} covering the vicinity of the minimum. The optimal value was then obtained by spline interpolation. For most dyes the optimal ω lies around 0.2 Bohr^{-1} in the gas phase and approximately 5% (with QM/MM) to 10% (with QM/MM+IEDRF) higher in n-hexane; the values for each chromophore are listed in table S1. A typical tuning curve $J^2(\omega)$ is shown in Fig. S1. It is not surprising that the QM/MM tuning curve differs little from the gas phase curve. The QM/MM embedding scheme only adds electrostatic interaction and Pauli repulsion, but the MM point charges on n-hexane are very small.

Table S1: Optimal range-separation parameters for Pasman dyes in the gas phase and in n-hexane (with either QM/MM or QM/MM+IEDRF embedding schemes for a single snapshot).

chromophore	$\omega_{\text{opt}}^{\text{vacuum}}$ Bohr^{-1}	$\omega_{\text{opt}}^{\text{QM/MM}}$ (n-hexane) Bohr^{-1}	$\omega_{\text{opt}}^{\text{QM/MM-IEDRF}}$ (n-hexane) Bohr^{-1}
1a	0.18	0.19	0.20
1b	0.17	0.18	0.20
1c	0.18	0.19	0.20
2a	0.19	0.20	0.22
3b	0.19	0.21	0.22
4c	0.19	0.19	0.20
5b	0.19	0.19	0.22
5c	0.20	0.20	0.22
6a	0.22	0.22	0.23
6b	0.20	0.21	0.23
6c	0.21	0.22	0.24
6d	0.20	0.21	0.22
7c	0.21	0.21	0.23
8c	0.21	0.21	0.23

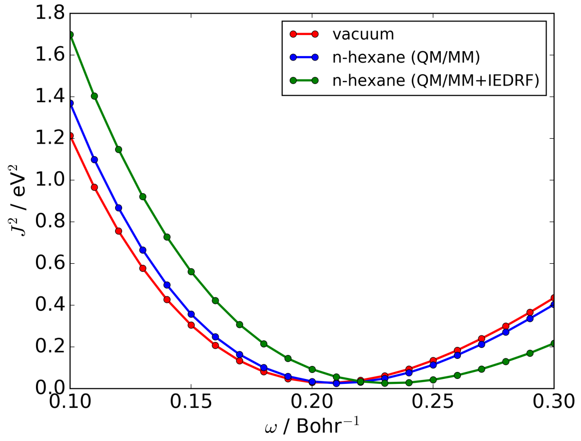

Figure S1: Tuning curve for dye 7c in vacuum and n-hexane.

4 Locally Excited States

The energies of the locally excited states are compared in Fig. S2. When comparing theoretical and experimental excitation energies, we are faced with the problem that the TD-DFT spectrum contains more excited states than there are peaks in the absorption spectrum in the energy range of interest. We select the lowest bright state closest to the experimental LE peak. With the exception of 7c and 9c, the QM/MM-IEDRF embedding scheme shifts the excitation energies of the LE state to slightly lower energies, which can be attributed to the dispersion interaction with the polarizable environment which is larger in the excited state than in the ground state. As a result, the overall agreement with experiment is improved.

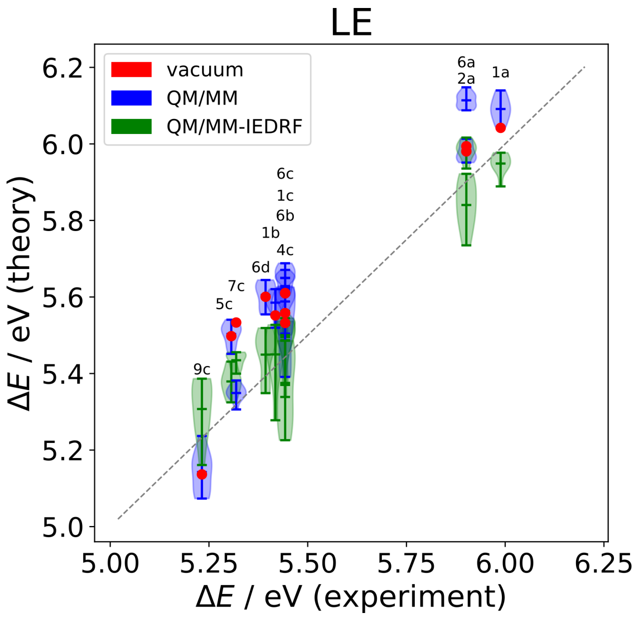

Figure S2: Correlation between experimental absorption band maxima of the LE state (experiment) and the vertical TD-ω_{opt}PBEh/aug-cc-pVDZ excitation energy of the closest bright state (theory) with different embedding schemes: isolated chromophore in gas phase (vacuum), electrostatic (QM/MM) and electrostatic+polarizable embedding (QM/MM-IEDRF). Violin plots indicate the distribution of energies among the 10 snapshots. The diagonal dashed line indicates a perfect correlation.

5 Dyes Excluded from the Analysis

The dyes 3b, 5b and 8c were excluded from the analysis, since we were either not sure about the character of the lowest electronic state, or the experimental assignments and energies were unreliable. For the dyes 3b and 8c, Pasman et al. observed only a single peak, which they assign to a local excitation. However, the TD-DFT calculations predict the lowest states to have charge transfer character. 3d can occur in two conformations which differ by the orientation of the methyl group in the 1-methylpiperidine donor group (see Fig. S3). In the gas phase, the conformation with an equatorial methyl group is more stable by 2.3 kcal/mol than the axial one. In the axial conformer the non-bonding nitrogen orbital on the donor is hybridized with the π orbital of the acceptor (Fig. S3b), so that the $n \rightarrow \pi^*$ excitation acquires a relatively large oscillator strength of 0.18 a.u. In the equatorial conformer, on the other hand, the lowest excitation is dark ((Fig. S3a). Based on these calculations the single

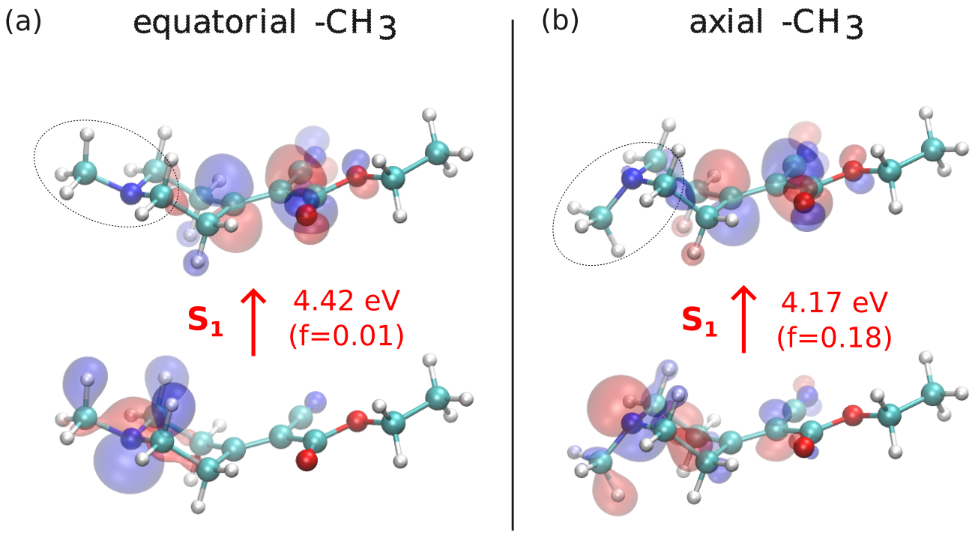

Figure S3: HOMO and LUMO orbitals (isovalue=0.05) of the S_1 transition of the two conformers of dye 3b.

peak seen in the experimental spectra could originate from the axial conformer and just be a CT state. However, this assignment conflicts with the energy of the absorption maximum of 5.2 eV, which is much higher than the computed CT energies of 4.4 eV (equatorial -CH_3) and 4.2 eV (axial -CH_3). In the TD-ωPBEh/aug-cc-pVDZ spectra the local excitations lie at 5.5 eV (equatorial -CH_3) and 5.8 eV (axial -CH_3), which agrees much better with the observed absorption maximum.

8c is a stereoisomer of 7c, in which the donor group forms an angle of 90° with the acceptor group. The transition density of the $n\pi^*$ state is zero and the CT state is dark (see Fig. S4). This state is not observed experimentally, either, which could be due to its low oscillator strength.

Finally, the CT energies of dyes 5b and 6c are both listed as 34500 cm^{-1} (4.28 eV) in table I of Pasman et al., which could be a coincidence or a typo. This energy agrees reasonably well with the TD-ωPBEh/aug-cc-pVDZ estimate for the CT state of 6c (4.44 eV), but is too low when compared with the TD-DFT estimate for 5b (4.69 eV).

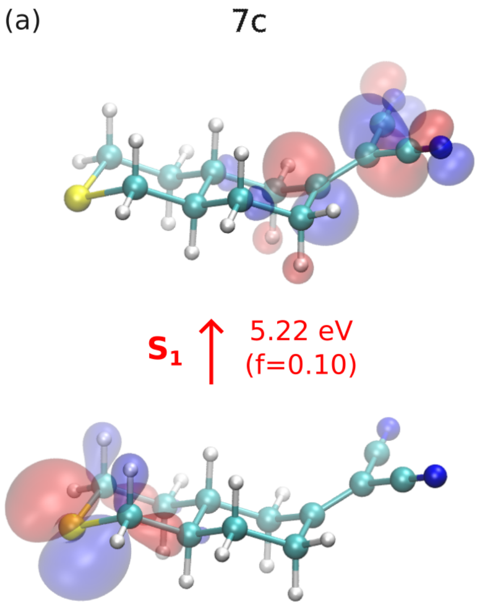

Figure S4: HOMO and LUMO orbitals (isovalue=0.05) of the S_1 transition of the dyes 7c and 8c.

References

(1) Applequist, J.; Carl, J. R.; Fung, K.-K. Atom dipole interaction model for molecular polarizability. Application to polyatomic molecules and determination of atom polarizabilities. *J. Am. Chem. Soc.* **1972**, *94*, 2952–2960.

(2) Thole, B. T. Molecular polarizabilities calculated with a modified dipole interaction. *Chem. Phys.* **1981**, *59*, 341–350.

(3) Yamaguchi, Y.; Goddard, J. *A New Dimension to Quantum Chemistry: Analytic Derivative Methods in Ab Initio Molecular Electronic Structure Theory*; Oxford University Press, 1994.

(4) Rohrdanz, M. A.; Martins, K. M.; Herbert, J. M. A long-range-corrected density functional that performs well for both ground-state properties and time-dependent density functional theory excitation energies, including charge-transfer excited states. *J. Chem. Phys.* **2009**, *130*, 054112.

(5) Foster, M. E.; Wong, B. M. Nonempirically tuned range-separated DFT accurately predicts both fundamental and excitation gaps in DNA and RNA nucleobases. *J. Chem. Theory Comput.* **2012**, *8*, 2682–2687.

(6) Kronik, L.; Stein, T.; Refaely-Abramson, S.; Baer, R. Excitation gaps of finite-sized systems from optimally tuned range-separated hybrid functionals. *J. Chem. Theory Comput.* **2012**, *8*, 1515–1531.
